# Buckwheat Flavonoids Modulate Inflammation in RAW 264.7 Macrophages at Physiologically Relevant Concentrations via the LPS/COX‑2 Pathway

**DOI:** 10.1021/acs.jafc.6c02109

**Published:** 2026-03-13

**Authors:** Diego José López-Cánovas, Antonio Vico-Padilla, Danuta Zielińska, Sabrina Poveda-Lora, Silvia Navarro-Orcajada, David López-Martínez, Diana García-Moreno, María Ángeles Ávila-Gálvez, Beatriz Garay-Mayol, José E. Yuste, Fernando Vallejo, Juan Carlos Espín, Antonio González-Sarrías, Henryk Zielinski, Juan Antonio Giménez-Bastida

**Affiliations:** a Quality, Safety & Bioactivity Plant Foods, Food Science & Technology Dep., CEBAS-CSIC, Murcia 30100, Spain; b Food and Health Laboratory, Food Science and Technology Dep., CEBAS-CSIC, Murcia 30100, Spain; c Department of Chemistry, 49674 University of Warmia and Mazury, Olsztyn 10-721, Poland; d Dep. Biochemistry and Molecular Biology-A, Faculty of Veterinary, University of Murcia, Murcia 30100, Spain; e Center for Biomedical Research in Rare Diseases Network (CIBERER), Carlos III Health Institute, Madrid 28029, Spain; f Biomedical Research Institute of Murcia (IMIB)-Pascual Parrilla, Murcia 30120, Spain; g Metabolomics Platform, CEBAS-CSIC, Murcia 30100, Spain; h InLife Institute of Animal Reproduction and Food Research, Team of Chemistry and Biodynamics of Food, Polish Academy of Sciences, Władysława Trylińskiego 18, Olsztyn 10-683, Poland

**Keywords:** eicosanoids, (poly)phenols, hPGDS, QTOF, Fagopyrum, flavones, flavonols

## Abstract

Buckwheat (BW) is recognized as a functional food with antioxidant and anti-inflammatory properties. BW (poly)­phenols are important bioactive compounds associated with these benefits, although their therapeutic role remains elusive. We used a multidisciplinary approach to identify the bioactive flavonoids of BW and their molecular mechanisms. Physiologically relevant concentrations of luteolin (Lute), quercetin (Quer), apigenin (Api), and kaempferol (Kaem) were effective in reducing prostaglandin (PG)­E_2_ and PGD_2_ biosynthesis in LPS-activated macrophages by acting at distinct branch points of the LPS/COX-2 pathway. Lute, Api, and Kaem reduced COX-2 levels, whereas Quer exerted the opposite effect. Lute and Kaem inhibited Ikkβ phosphorylation, while TLR4 was identified as a flavonoid's target. PGE_2_ and PGD_2_ reductions were independent of COX-2 modulation and correlated with hematopoietic prostaglandin D synthase (hPGDS) inhibition (exerted by Lute and Quer). These findings offer a new perspective on the LPS/COX-2 pathway as a target of BW-derived products to address inflammation-related diseases.

## Graphical abstract

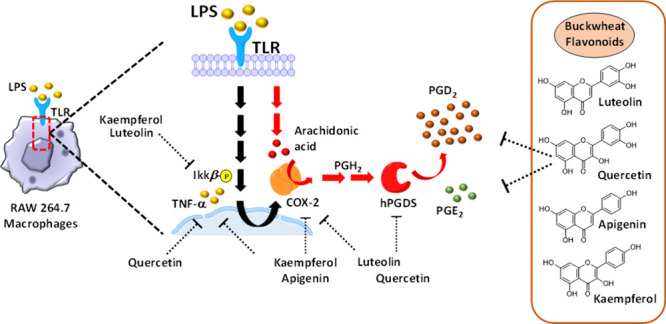

## 1 Introduction

Buckwheat (BW), a gluten-free pseudocereal of the *Polygonaceae* family (genus *Fagopyrum*), encompasses a range of species with common (*Fagopyrum esculentum*) and Tartary (*Fagopyrum tataricum*) BW as the most relevant varieties. Its integration into modern agricultural systems constitutes an attractive strategy for enhancing nutritional security and overall well-being.^1^ BW is a highly appreciated natural product recognized as an attractive functional food thanks to its high nutritional value and health-promoting compounds.^2^ It is mainly commercially available as BW seeds (raw groats), BW-enriched products (BW bread, snacks), or sprouts.^3^ The anti-inflammatory and antioxidant health-related effects in humans associated with BW consumption in its different forms^4–9^ have spurred notable interest among the food science community to identify its bioactive compounds with therapeutic potential.

Inflammation-related animal models fed BW ethanol extracts showed anti-inflammatory effects by modulating key markers.^10,11^ The chromatographic characterization of the extracts together with the use of diets enriched in BW flavonoids (i.e., rutin and quercetin (Quer)), underscores the role of these molecules as constituents that most likely mediate the anti-inflammatory effects described.^10,12,13^ Nonetheless, it is difficult to determine the specific role of these molecules in the effects observed. Notable differences in BW (poly)­phenol composition, linked to variety (e.g., dissimilar rutin levels in tartary vs common BW), growth conditions, product type tested (honey, bread, noodles, groats, or sprouts), processing method (gluten-free vs BW-enriched), and plant part (flowers, leaves, and stems),^3,14–16^ create uncertainty about their specific role. Overcoming this barrier requires pinpointing the crucial bioactive molecule(s), defining the conditions for actionable results, and deepening investigation of relevant cellular and molecular targets.^17–19^

Macrophages are an integral element of the immune system, and their regulation portrays an attractive therapeutic target.^20^ RAW 264.7 macrophages are a widely used cellular model to investigate the anti-inflammatory effects of BW.^21–24^ Mechanistic analyses conducted in these studies highlight the LPS/COX-2 pathway as a key target of this natural product via inhibition of prostaglandin (PG)­E_2_ biosynthesis, COX-2 expression, and NF-κB activation.^21–23^ Further important branch points of this path, usually overlooked, include the interaction between TLR4 and (poly)­phenols, effects on COX-2 and hematopoietic prostaglandin D synthase (hPGDS) enzymatic activity, inhibition of the inhibitor of nuclear factor kappa-B kinase subunit beta (Ikkβ) phosphorylation as a mechanism related to NF-κB modulation, and PGD_2_ formation. This lack of knowledge hinders our understanding of the identities of bioactive compounds and their underlying molecular mechanisms, thereby limiting scientific progress in developing BW-derived functional foods tailored to promote well-being.

Consequently, we report here an investigation of the anti-inflammatory effects of BW (poly)­phenols (phenolic acids and flavonoids at relevant *in vivo* concentrations) by studying common and less-explored mechanisms within the LPS/COX-2 pathway. Using LPS-activated RAW 264.7 macrophages as a cellular model, we specifically explored the effects on: (i) PGs biosynthesis, (ii) COX-2 level, (iii) modulation of Ikkβ phosphorylation, (iv) COX-2/hPGDS activity, and (iv) *in silico* simulation of the interaction between BW flavonoids and key targets. We also assayed the antioxidant/reducing activity of BW (poly)­phenols, their cellular phase-II metabolism, and their effects on fundamental pro-inflammatory cytokines (e.g., TNF-α) to provide a compelling picture of their impact on the LPS/COX-2 axis.

Considering the previous evidence reported,^21–23^ we describe here uncharacterized molecular mechanisms by which BW (poly)­phenols modulate inflammation in activated macrophages, redefining our comprehension of the LPS/COX-2 pathway as a critical target of BW (poly)­phenols, including its flavonoids.

## 2 Materials and Methods

### 2.1 Materials

Methanol (MeOH), acetic acid (LC-MS grade), sodium acetate, lipopolysaccharide (LPS), horseradish peroxidase (HRP), hydrogen peroxide (H_2_O_2_), porcine hematin (H2381), and phosphate-buffered saline (PBS) were purchased from Merck (KGaA, Darmstadt, Germany). 2,2′-Azino-bis­(3-ethylbenzothiazoline-6-sulfonic acid) diammonium salt (ABTS), 2,2-diphenyl-1-picrylhydrazyl (DPPH), 6-hydroxy-2,5,7,8-tetramethylchroman-2-carboxylic acid (Trolox), and magnesium chloride (MgCl_2_) were obtained from Sigma (Sigma Chemical Co., St. Louis, MO, USA). The standards (phenolic acids, flavonoids) used were provided by Sigma Chemical Co. (St. Louis, MO, USA), Supelco (Bellefonte, PA, USA), and Phyproof (Vestenbergsgreuth, Germany) as follows: Rutin (PHL89270 ≥95%; Phyproof), epicatechin (E1753 ≥90%; Sigma-Aldrich), apigenin (10798 ≥95%; Sigma-Aldrich), quercetin (PHR1488; Supelco), luteolin (L9283 ≥98%; Sigma-Aldrich), kaempferol (60010 ≥97%; Sigma-Aldrich), vanillic acid (H36001 ≥97%; Sigma-Aldrich), protocatechuic acid (08992 ≥97%; Supelco), caffeic acid (205546 ≥95%; Sigma-Aldrich), syringic acid (S8005 ≥98%; Sigma-Aldrich), sinapic acid (D7927 ≥98%; Sigma-Aldrich), ferulic acid (128708 99%; Sigma-Aldrich), p-coumaric acid (PHL89498 ≥95.0%; Phyproof), and *trans*-cinnamic acid (C80857 99%; Sigma-Aldrich). The Milli-Q system (Millipore, Bedford, USA) was used for water purification. Arachidonic acid (AA), prostaglandin (PG)-E_2_, PGD_2_, PGE_2_-d_4_ (deuterated internal standard), prostaglandin D synthase (hematopoietic-type, human recombinant; hPGDS), aspirin (ASA), prostaglandin D synthase (hematopoietic-type) inhibitor I (INH-1), reduced glutathione (GSH), and phenylmethylsulfonyl fluoride (PMSF) were purchased from Cayman Chemical (Ann Arbor, MI, USA) and obtained through Vitro S.A. (Madrid, Spain) and Labclinics (Barcelona, Spain) as official distributors. Ammonium acetate buffer (NH_4_OAc) and dimethyl sulfoxide (DMSO) were obtained from Panreac (Barcelona, Spain). Recombinant human COX-2 (hCOX-2) was expressed in Sf9 cells and purified as described elsewhere^25,26^ with modifications detailed in an optimized protocol (10.5281/zenodo.18004428).

### 2.2 Dosage Information for the Cellular Experiments

BW phenolic acids and flavonoids (Figure 1) were diluted in DMSO (10 mM stock solution). RAW264.7 macrophages were activated with 10 μg/mL LPS (diluted in PBS) and treated with these natural compounds at concentrations between 15 and 0.1 μM (≤0.5% DMSO, v/v). Vehicle-treated cells (PBS and DMSO at equivalent concentrations) were used as the controls. These concentrations of BW phenolic compounds and flavonoids were similar to those detected *in vivo* at the intestinal level^27,28^ and lacked cytotoxic effects under the conditions of our study (Figure S1).

**Figure 1:**
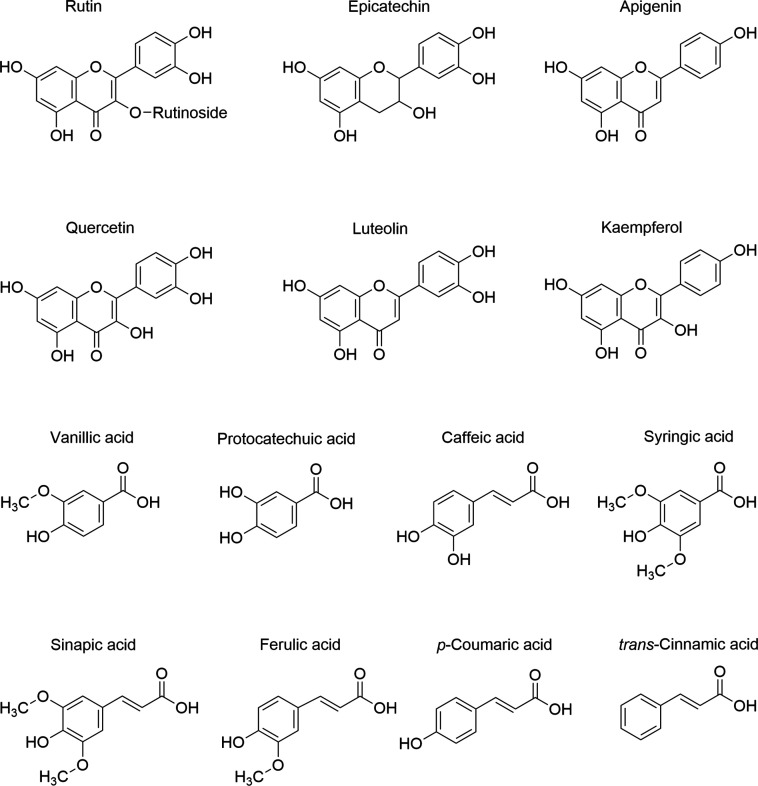
Chemical structures of relevant BW phenolic acids and flavonoids.

### 2.3 Cell Culture: RAW 264.7 Macrophages and THP-1 Monocytes

Murine macrophages RAW 264.7 obtained from the ATCC collection (TIB-71; Manassas, VA, USA) were cultured in phenol red-free DMEM enriched with 4.5 g/L glucose and 10% (v/v) fetal bovine serum (FBS). Complete growth medium also included 2 mM glutamine and 1 mM penicillin/streptomycin as supplements. THP-1 monocytes, obtained from the European Collection of Authenticated Cell Cultures (ECACC, Salisbury, U.K.), were grown in a RPMI 1640 culture medium enriched with 10% (v/v) FBS and 2 mM -glutamine and supplemented with antibiotics such as penicillin (100 U/mL) and streptomycin (100 μg/mL) (Gibco, Invitrogen S.A., Barcelona, Spain). The handling conditions for both cell lines are described in the Supporting Information.

### 2.4 RAW 264.7 Macrophages Treatment and Analysis of PGs Biosynthesis and (Poly)­phenols Metabolism Using UPLC-QTOF-MS

The cells were seeded at 10,000 cells/cm^2^ in 6-well plates and grown for 5 days as described above to evaluate the effects of BW phenolics and flavonoids. Next, the medium was removed, the cell surface was washed with PBS, and the cells were incubated in a FBS-deprived medium for 24 h to minimize the impact of FBS on COX-2.^29^ In a first set of experiments, the cells were cotreated with 15 μM BW phenolic acids or flavonoids (Figure 1) and 10 μg/mL LPS for 4 h. In a second group of assays, lower concentrations (5, 1, and 0.1 μM) were tested for those compounds that exerted significant inhibition at 15 μM.

The culture media obtained from different treatments were stored at −80 °C until their analysis. Extraction of metabolites from culture medium followed a procedure described elsewhere;^30^ a brief description is included in Supplementary methods (Supporting Information). We analyzed the samples using an Agilent 1290 Infinity UPLC-ESI system connected to a 6550 Accurate-Mass QTOF (Quadrupole-Time-Of-Flight) from Agilent Technologies (Waldbronn, Germany). A Poroshell 120 EC-C18 2.7 mm column (3.0 × 100 mm; Agilent Technologies, Santa Clara, CA, USA) was used for the separation of the PGs and the phenolic compounds (precursors and derived metabolites) in each sample (5 μL injection). Water (mobile phase—A) and acetonitrile (mobile phase—B), both acidified with 0.1% (v/v) formic acid, were used at a flow rate of 0.4 mL/min. The samples, analyzed in negative ion mode, were separated using a linear gradient (solvent A used as a reference) as follows: 0 min—99%; 10 min—50%; 14 min—15%; 17 min—0%; 19 min—99% (initial conditions); 24 min—99%. Spectra acquisition used a *m*/*z* range between 100 and 1100 at a scan rate of 1.5 scan/s. Optimal electrospray ionization parameters using nitrogen as nebulizer gas were: capillary voltage was 3500 V; nitrogen temperature and flow were 280 °C and 11 l/min, respectively. The nebulizer pressure was set at 45 psig, and the nozzle voltage was 500 V. The MassHunter Qualitative Analysis software (version B.08.00, Agilent) was selected for data analysis. We used pure standards (see Section 2.1) to accurately identify and quantify the PGs and (poly)­phenol-derived metabolites released into the culture medium by LPS-activated RAW 264.7 cells. When pure standards were unavailable, we used a previously reported strategy^31^ that combines parameters such as the isotopic pattern, molecular formula, elution order, accurate mass (extraction window set at 0.01 *m*/*z*), and score to identify metabolites (Table 1 and Table S1).

**Figure 2:**
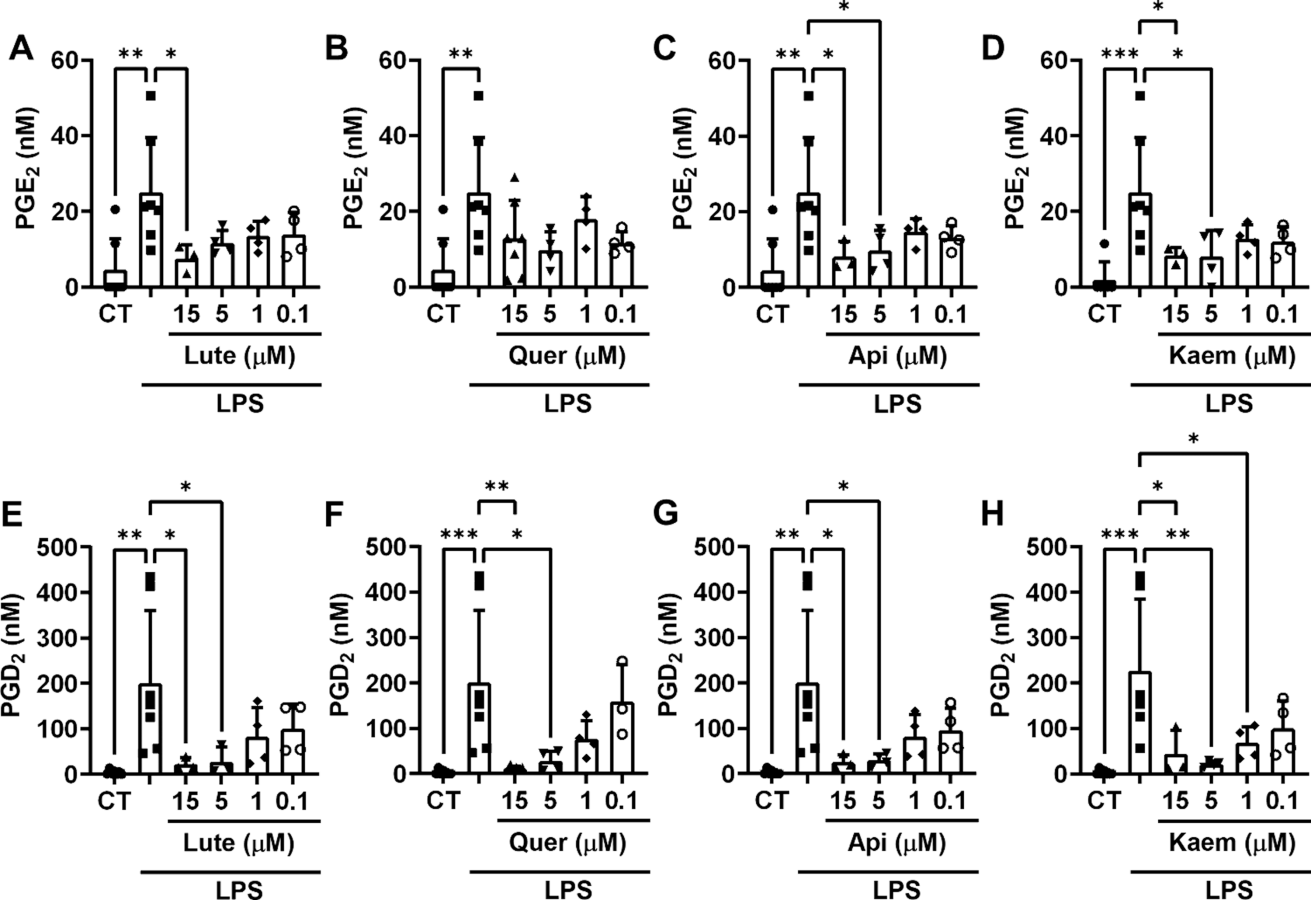
Dose-dependent effect of Lute, Quer, Api, and Kaem on COX-2-derived PGE_2_ (A–D) and PGD_2_ (E–H) in culture media obtained from RAW 264.7 macrophages stimulated with 10 μg/mL LPS for 4 h. UPLC-QTOF analysis and quantification of the PGE_2_ and PGD_2_ concentrations in the culture medium of the different treatments. The bar graphs, displayed as mean ± SD, illustrate results from independent experiments (*n* = 3–7). ANOVA analysis and Holm–Sidak *post hoc* test were used to determine statistically significant differences: *, *p* < 0.05; **, *p* < 0.01; ***, *p* < 0.001 versus LPS-stimulated RAW 264.7 macrophages.

**Table 1:** Metabolism of BW Phenolic Acids and Flavonoids by LPS-Activated RAW 264.7 Macrophages after 4 h of Treatment^d^

compound	retention time (min)	mean ± SD
**rutin treatment**		
**rutin**	5.976	11 ± 4.8^a^
**epicatechin treatment**		
epicatechin sulfate (peak 1)	4.181	34,262 ± 9798^b^
epicatechin sulfate (peak 2)	4.678	n.q.^c^
methyl epicatechin glucuronide	5.163	108,577 ± 20,788^b^
**epicatechin**	5.231	1.2 ± 0.9^a^
methyl epicatechin (peak 1)	6.077	685,055 ± 112,522^b^
methyl epicatechin (peak 2)	6.461	67,571 ± 7461^b^
**Lute treatment**		
Lute glucuronide (peak 1)	6.316	76,140 ± 107,592^b^
Lute sulfate (peak 1)	6.655	61,903 ± 29,651^b^
Lute sulfate (peak 2)	6.847	2,835,389 ± 4,265,759^b^
Lute glucuronide (peak 2)	6.982	130,911 ± 124,393^b^
methyl Lute glucuronide (peak 1)	7.095	658,008 ± 999,056^b^
Lute glucuronide (peak 3)	7.186	463,531 ± 302,588^b^
methyl Lute glucuronide (peak 2)	7.220	323,818 ± 350,919^b^
methyl Lute glucuronide (peak 3)	7.366	3,945,610 ± 736,696^b^
**Lute**	8.450	1.4 ± 1.2^a^
methyl Lute	9.621	72,049,622 ± 57,293,522^b^
**Quer treatment**		
Quer glucuronide (peak 1)	6.224	493,048 ± 735,268^b^
methyl Quer glucuronide (peak 1)	6.878	859,026 ± 942,764^b^
Quer glucuronide (peak 2)	7.003	349,499 ± 498,398^b^
methyl Quer glucuronide (peak 2)	7.183	115,718 ± 92,160^b^
methyl Quer glucuronide (peak 3)	7.296	189,864 ± 230,142^b^
methyl Quer glucuronide (peak 4)	7.432	708,714 ± 865,551^b^
**Quer**	8.527	4.9 ± 2.8^a^
**Api treatment**		
Api glucuronide (peak 1)	6.980	3,943,029 ± 5,087,256^b^
Api glucuronide (peak 2)	7.081	645,388 ± 779,741^b^
Api sulfate	7.713	108,442 ± 56,879^b^
**Api**	9.418	8.1 ± 4.5^a^
methyl Api	11.777	322,890 ± 231,164^b^
**Kaem treatment**		
Kaem glucuronide	6.750	4,918,071 ± 4,845,630^b^
**Kaem**	9.629	4.9 ± 3.1^a^
methyl Kaem sulfate	9.978	247,737 ± 93,562^b^
**vanillic acid treatment**		
vanillic acid glucuronide	4.696	1,150,511 ± 400,884^b^
**vanillic acid**	5.192	8.9 ± 1.9^a^
**protocatechuic acid treatment**		
protocatechuic acid sulfate	1.820	734,081 ± 301,697^b^
protocatechuic acid glucuronide	3.150	n.q.^c^
**protocatechuic acid**	3.865	16 ± 4.1^a^
**caffeic acid treatment**		
**caffeic acid**	5.180	15 ± 3.8^a^
**syringic acid treatment**		
syringic acid sulfate	4.160	51,923 ± 10,930^b^
**syringic acid**	5.280	16 ± 3.2^a^
**sinapic acid treatment**		
sinapic acid glucuronide	5.190	28,886 ± 17,204^b^
**sinapic acid**	6.730	6.8 ± 1.2^a^
**ferulic acid treatment**		
ferulic acid glucuronide	5.029	26,197 ± 10,493^b^
**ferulic acid**	6.507	14 ± 3.5^a^
***p*** **-coumaric** **acid treatment**		
***p*** **-coumaric** **acid**	6.152	13 ± 3.2^a^
***trans*** **-cinnamic acid treatment**		
***trans*** **-cinnamic acid**	9.099	12 ± 3.1^a^

a Pure standards were used to quantify free forms, which are expressed as μM.

b Conjugated metabolites were tentatively identified in the absence of pure standards and are reported as peak areas obtained from extracted ion chromatograms (EIC).

c n.q. = detected but not quantifiable (signal-to-noise ratio, S/N < 10).

d Data are expressed as mean ± SD from three independent analyses (*n* = 3).

### 2.5 Western Blot

The cold RIPA buffer supplemented with phosphatase and protease inhibitors (Roche, Mannheim, Germany) was used to extract the cellular proteins from the samples obtained in the previous assay. Equal protein amounts (30 μg), quantified by the DC protein assay (Bio-Rad, Hercules, CA, USA), were loaded in a 10% SDS-polyacrylamide gel, transferred to nitrocellulose membranes (GE Healthcare, Buckinghamshire, UK), and incubated with primary COX-2 (74 kDa; D5H5, #12282) or β-actin (45 kDa; #4967) from Cell Signaling (MA, USA) at a dilution of 1:1000 and with secondary antirabbit antibody (#7074) at a dilution 1:5000. Band intensity quantification using ImageJ version 1.53k (NIH, USA) allowed the comparison between the treatments and the LPS-activated macrophages. β-Actin served as a loading control to normalize the COX-2 level. Figure 3 illustrates the data obtained from three independent experiments (*n* = 3).

**Figure 3:**
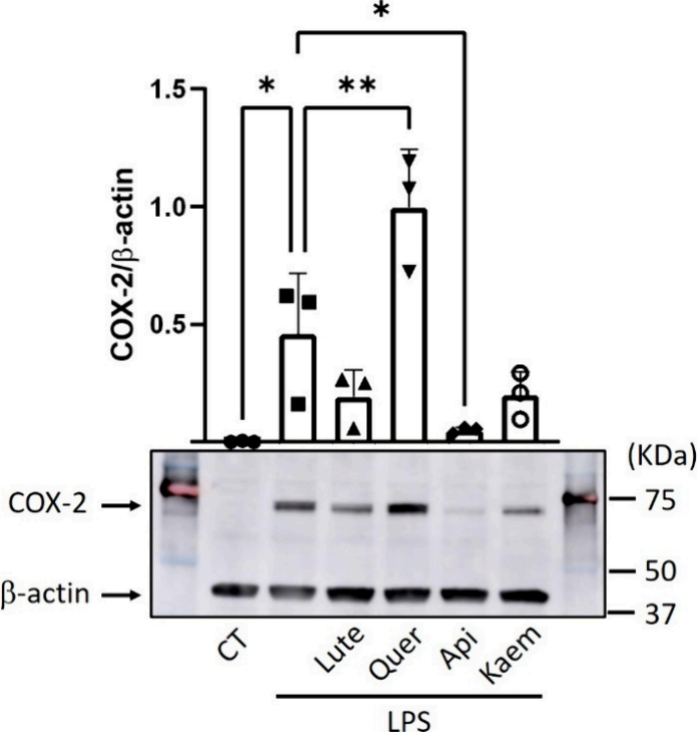
Effect of BW flavonoids on the COX-2 protein levels. RAW 264.7 macrophages were cotreated with 15 μM BW flavonoids (Lut, Quer, Api, Kaem; 0.5% DMSO v/v) and 10 μg/mL LPS (0.5% DMSO v/v) for 4 h. Unstimulated cells (CT) treated with an equivalent dose of DMSO (0.5% v/v) were used as a negative control. Western blot analysis of the cellular protein involved intensity quantification of the COX-2 bands and normalization to the loading control, β-actin. The results shown as mean ± SD come from three independent experiments (*n* = 3). ANOVA analysis and Holm–Sidak *post hoc* test were used to determine statistically significant differences: *p < 0.05 and **p < 0.01 versus LPS-stimulated RAW 264.7 cells.

### 2.6 ELISA Assays—TNF-α and Ikkβ Phosphorylation

RAW 264.7 macrophages incubated in a FBS-deprived medium for 24 h were cotreated with 15 μM BW flavonoids and 100 ng/mL LPS for 4 h. The culture medium and cellular protein (obtained using RIPA buffer containing phosphatase and protease inhibitors) were collected and stored at −80 °C until their analysis. TNF-α was determined by ELISA (PeproTech, Thermo Fisher; Waltham, MA, USA), and its concentration was measured using a microplate reader (Infinite M2000, TECAN, Grödig, Austria). TNF-α concentration was normalized to total protein (quantified by DC protein assay) and expressed as the mean ± SD from four independent experiments (*n* = 4). THP-1 monocytes were incubated in an FBS-deprived RPMI 1640 medium for 1 h, followed by the cotreatment with 15 μM BW flavonoids and 10 μg/mL LPS for 30 min (time point optimized as shown in Figure S3). Ikkβ phosphorylation at Ser177/181 was performed following the manufacturer’s instructions for a PathScan Phospho-Ikkβ sandwich ELISA kit (#7080) from Cell Signaling Technology (MA, USA).

### 2.7 Measurement of the Reducing Potency, Antioxidant, and Chelating Activities

For cyclic voltammetry (CV) experiments, we used standard solutions of phenolic acids and flavonoids (500 μM) or Trolox (from 0.1 to 2.5 mM) in 0.2 M sodium acetate-acetic buffer (pH = 4.5; 80% v/v MeOH) at a 1:1 (v/v) ratio as described elsewhere.^37,38^ The cyclic voltammograms generated by a potentiostat/galvanostat G 750 (Gamry Ins., USA) came from experiments using a range from −100 to +1300 mV. For the remaining experiments, phenolic acids and flavonoids were diluted in MeOH to 1 mM, and their concentrations were confirmed according to Franke et al.^39^ These stock solutions were the basis of the experiments to determine the antioxidant activity (DPPH radicals assay),^40^ the ferric-reducing/antioxidant power (FRAP),^41^ and the ferrous ions chelating activity (inhibition at pH 7.5 of the formation of Fe^2+^—ferrozine complex)^42^ following established methodologies. We used a temperature-controlled UV–vis spectrophotometer (UV-1601PC with CPS-Controller Shimadzu, Japan), and the results were expressed as mM Trolox equivalents. The data obtained are the result of six independent experiments (*n* = 6).

### 2.8 HRP/H_2_O_2_ Oxidation

HRP-catalyzed oxidation of 50 μM Lute, Quer, Api, and Kaem was conducted in 20 mM NH_4_OAc buffer (pH = 6.8) containing 200 μM H_2_O_2_ and HRP (6 mU/mL) at room temperature. UV–vis spectra (220–800 nm) were recorded sequentially in a JASCO V-630 spectrophotometer (Tokyo, Japan). The reaction was initiated by adding each flavonoid in the presence of H_2_O_2_ (time 0, first cycle), followed immediately by HRP addition, and the spectra were recorded every 2.5 min for a total of 12.5 min to monitor spectral changes over time.

### 2.9 COX-2 and COX-2/hPGDS Incubations

COX-2 enzymatic incubations were carried out in 100 μL of 100 mM NH_4_OAc buffer (pH 8) supplemented with 2 μM hematin, 500 μM phenol, and 100 nM recombinant human COX-2 (hCOX-2). For the COX-2/hPGDS incubations, the same reaction mixture also contained 500 μM GSH, 0.5 M MgCl_2_, and 0.5 μM hPGDS. To test the effect of BW flavonoids (Lute, Quer, Api, and Kaem), we added the compounds (15 μM final concentration) to the hCOX-2 or hCOX-2/hPGDS reaction mixture and incubated at room temperature for 1 h. Parallel incubations, under the same conditions, containing 1 mM ASA (COX-2 inhibitor) and 10 μM INH-1 (hPGDS inhibitor) for 1 h, served as inhibition controls. To trigger the reaction, we added 30 μM arachidonic acid (AA) and incubated the mixture for 15 min before stopping it by adding 2.5 μL of MeOH containing 0.2% (v/v) acetic acid (pH ∼3.5) supplemented with 10 μM PGE_2_-d_4_ (used as an internal standard). Metabolite extraction and data analysis followed the same protocol as described for cellular assays (see above and Supplementary methods (Supporting Information)).

### 2.10 Docking Analysis

The 3D structures of the target proteins were retrieved from the Protein Data Bank (PDB). The crystal structures used were: hPGDS (PDB ID: 2CVD, 1.45 Å), human Toll-like receptor 4/MD-2 (TLR4/MD-2; PDB ID: 2Z65, 2.70 Å), and murine cyclooxygenase-2 (COX-2; PDB ID: 3LN1, 2.40 Å). Cocrystallized ligands and nonessential water molecules were removed, and polar hydrogens were added. Protonation states were assigned according to the physiological pH (7.4). Although enzymatic incubations were conducted at pH = 8, protonation states were defined at pH = 7.4 since this value maintains most titratable residues in comparable states and minimizes computational artifacts associated with extreme pH adjustments in docking simulations.

Docking simulations were performed using the Docking (Vina) tool implemented in the Mcule platform (https://mcule.com). The search space was defined according to experimentally validated binding sites: for hPGDS, the catalytic pocket containing Trp104 and Arg14 (corresponding to Trp103 and Arg13 in the Mcule numbering);^32^ for TLR4/MD-2, the hydrophobic pocket centered on the sulfur atom of residue Cys133 with a 22 Å radius to encompass the entire binding cavity;^33^ and for COX-2, the active site pocket as defined in the 3LN1 structure.^34^ Ligand structures were obtained from PubChem in SMILES format, converted to 3D conformers, energy-minimized, and prepared by using the default Mcule pipeline. Known inhibitors were included as reference controls: HQL-79 for hPGDS,^32^ eritoran for TLR4/MD-2,^35^ and celecoxib for COX-2.^36^ Docking parameters were kept at default settings, and the lowest predicted binding free energies (Δ*G*_pred_, kcal/mol) were recorded. Binding poses and intermolecular interactions were further examined with a Discovery Studio Visualizer (BIOVIA). Noncovalent interactions, including hydrogen bonds, π–π stacking, π–σ, π–alkyl, alkyl, and salt bridges, were identified and systematically compared with those of the reference ligands.

### 2.11 Statistical Analysis

One-way analysis of variance (ANOVA) was used to analyze normally distributed data. The Pearson correlation coefficient and Holm–Sidak's *post hoc* test provided information about the linear relationship between the data and the statistically significant differences between the treatments, respectively. GraphPad Prism v. 10.4.1 for Windows (GraphPad Software, San Diego, CA, USA) was the software used for the graphical representation of the data and statistical analysis. Results are shown as mean ± SD (*n* = 3–7 independent replicates).

## 3 Results

### 3.1 BW Flavonoids (Lute, Quer, Api, and Kaem) Reduce the Biosynthesis of PGE_2_ and PGD_2_ in LPS-Stimulated RAW 264.7 Macrophages in a Dose-Dependent Manner

In an initial screening, we tested the capacity of BW phenolic acids and flavonoids to modulate the biosynthesis of PGE_2_ and PGD_2_ in LPS-activated RAW 264.7 macrophages. Cell activation with 10 μg/mL LPS for 4 h resulted in higher levels of PGE_2_ (25.1 ± 14.4 nM) and PGD_2_ (200.6 ± 160.0 nM) compared to unstimulated cells (4.6 ± 8.3 and 3.0 ± 5.5 nM, respectively). As expected, PGD_2_ was the major product formed (8-fold higher than PGE_2_) due to the increased expression of COX-2 and hPGDS in LPS-treated RAW264.7 ^43^ (Figure 2). BW phenolic acids, as well as the flavonoids rutin and epicatechin, were unable to modulate the PGE_2_ or PGD_2_ biosynthesis at 15 μM (Figure S2). Api and Kaem exerted a dose-dependent inhibition of both PGs (reaching a significant PGD_2_ inhibition at 1 μM with Kaem), whereas Lute and Quer showed a more efficient inhibition of PGD_2_ (significant at 15 and 5 μM) than PGE_2_ (only significant at 15 μM in Lute-treated cells) (Figure 2).

### 3.2 Metabolism of Phenolic Compounds

We next studied the metabolic transformations of BW phenolic acids and flavonoids in our cellular model to determine whether their conversions might be related to the differences observed in PG formation. RAW 264.7 cells were unable to metabolize rutin, the only glycoside investigated, as evidenced by the absence of new chromatographic peaks in the UPLC-QTOF-MS analysis. In contrast, an active phase-II metabolism of the remaining phenolic acids and flavonoids studied resulted in the formation of a range of (di)­conjugated molecules, yet the degree and type of metabolism seemed to be compound-specific. Phenolic acid metabolism resulted in the formation of glucuronides and/or sulfates, except for caffeic, *trans*-cinnamic, and *p*-coumaric acids (only detected in their free form). Otherwise, the flavonoids investigated were converted into glucuronide, sulfate, or methylglucuronide derivatives (Table 1).

### 3.3 BW Flavonoids Exert a Dissimilar Effect on COX-2 Levels in LPS-Activated RAW 264.7 Macrophages

We focused on BW flavonoids (Lute, Quer, Api, and Kaem) that modulate PG biosynthesis to explore associated mechanisms, such as COX-2 regulation. Western blot analysis (Figure 3) showed a reduction of the LPS-induced COX-2 level, in the presence of 15 μM Lute and Kaem (2.4-fold lower; *p* > 0.05), as well as Api (9-fold reduction; *p* < 0.05), in the macrophages. At the same concentration, Quer exerted the opposite effect, increasing the COX-2 level in a significant manner (2.2-fold increase; *p* < 0.01).

### 3.4 Lute and Kaem Inhibit Ikkβ Phosphorylation at Ser177/181

To assess whether the effect of BW flavonoids on COX-2 came from the modulation of the NF-κB route, we studied whether these natural products could regulate Ikkβ activation (critical in NF-κB activation) as an underlying mechanism. We used THP-1 human monocytes as a model to study the role of Ikkβ as a target of BW flavonoids. We first evaluated their inhibitory potency on Ikkβ phosphorylation, and we found that Lute and Kaem exerted a higher inhibition (≥25% inhibition) than Quer and Api (<15% inhibition) at 15 μM (Figure S3). Based on published data on the inhibition of NF-κ activity exerted by reference anti-inflammatory compounds, such as curcumin,^44^ we set 25% as a relevant inhibitory effect and performed additional experiments with Lute and Kaem to determine their significant inhibitory effects. As shown in Figure 4, both compounds significantly reduced the phosphorylation of Ser177/181 of Ikkβ, consistent with their effects on the COX-2 and PG levels.

**Figure 4:**
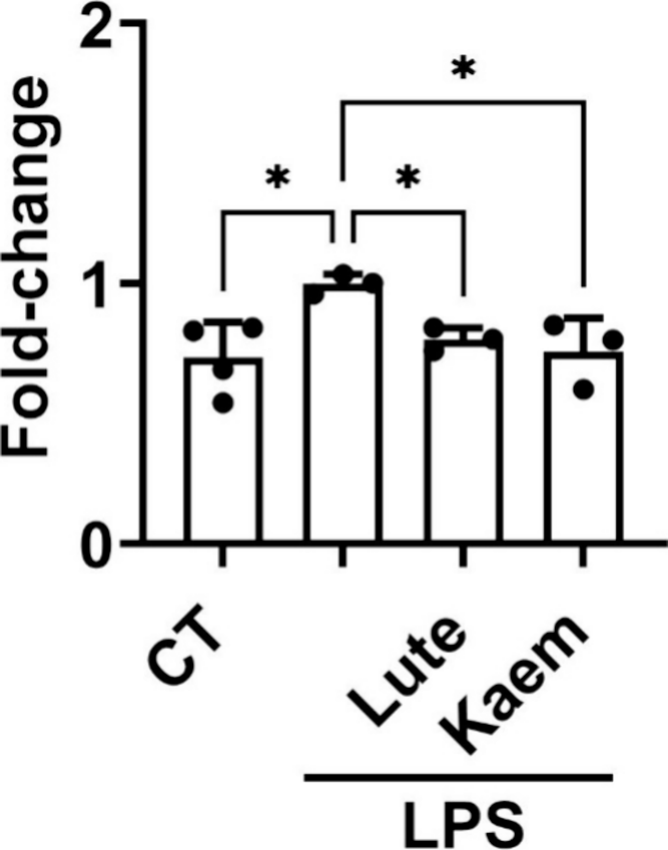
Inhibition of Ikkβ phosphorylation at Ser177/181. Cell protein for the ELISA assay was obtained from THP-1 monocytes treated with 15 μM Lute and Kaem for 1 h, followed by LPS stimulation (10 μg/mL) for 30 min. Protein phosphorylation is expressed as the mean ± SD of the ratio between the absorbance (450 nm) and protein concentration (nm/mL). The experiment was repeated at least three times (*n* = 3). ANOVA analysis and Holm–Sidak *post hoc* test allowed determination of statistically significant differences: *p < 0.05 versus LPS-stimulated THP-1 cells.

**Figure 5:**
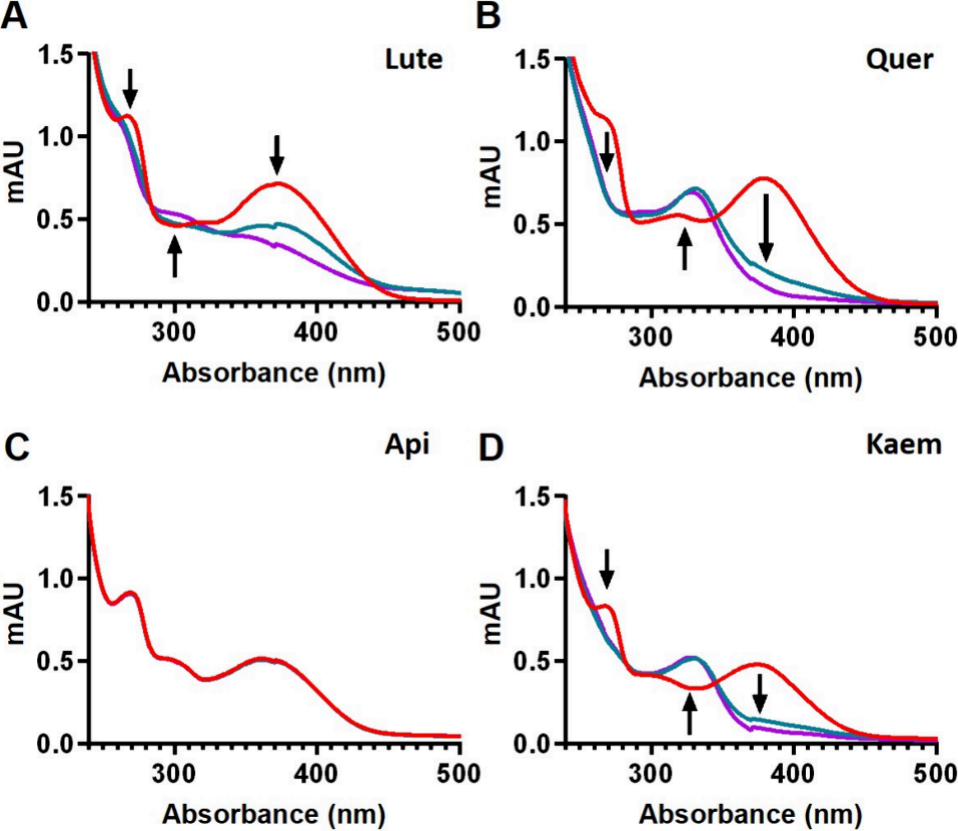
HRP/H_2_O_2_-catalyzed transformation of BW flavonoids, including (A) Lute, (B) Quer, (C) Api, and (D) Kaem. NH_4_OAc buffer (20 mM, pH = 6.8) together with H_2_O_2_ (50 μM) served as a blank. The reaction was initiated by adding 50 μM BW flavonoids (first scan; red spectrum), followed by the immediate addition of HRP (6 × 10^–6^ U/μL) to 1 mL of the reaction mixture. Repeated scans from 800 to 240 nm every 2 min for 12 min in a UV/vis spectrophotometer enabled the monitoring of spectral changes of each compound. Arrows indicate the direction of the spectral change at the recorded wavelengths.

**Figure 6:**
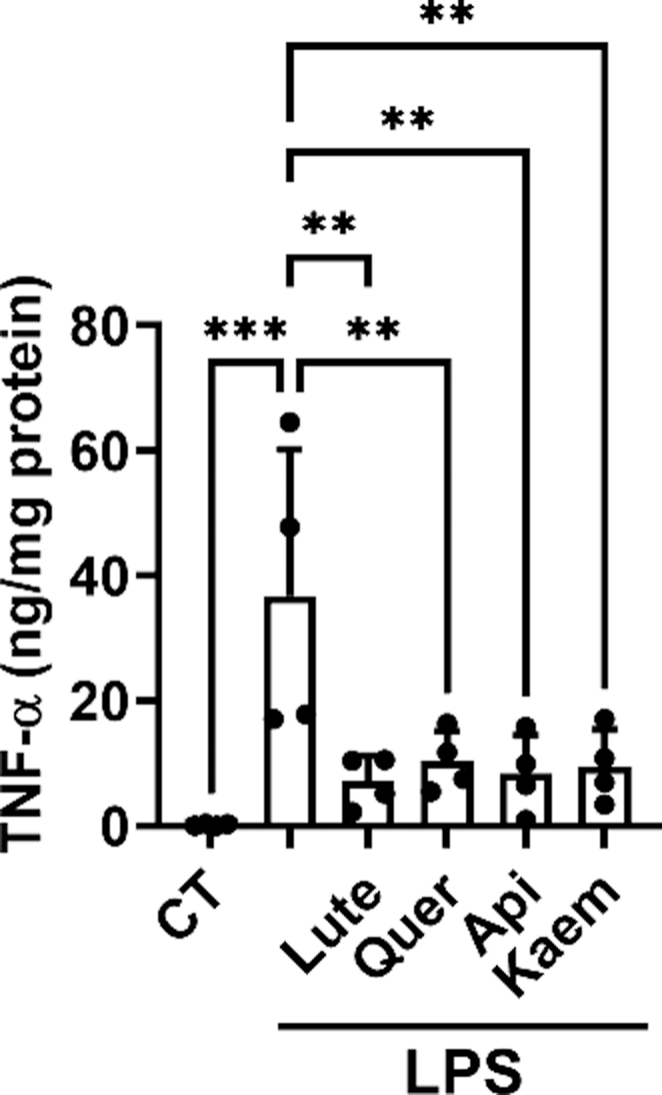
Effect of BW flavonoids on the biosynthesis of TNF-α in LPS-stimulated RAW 264.7 macrophages. The culture medium obtained from cells stimulated with 100 ng/mL LPS in the presence of 15 μM Lute, Quer, Api, and Kaem for 4 h was analyzed by ELISA. The data are shown as mean ± SD from four independent replicates (*n* = 4). ANOVA analyses followed by the Holm–Sidak *post hoc* test were used to determine statistically significant differences: **p < 0.01 and ***p < 0.001 versus LPS-treated cells.

**Figure 7:**
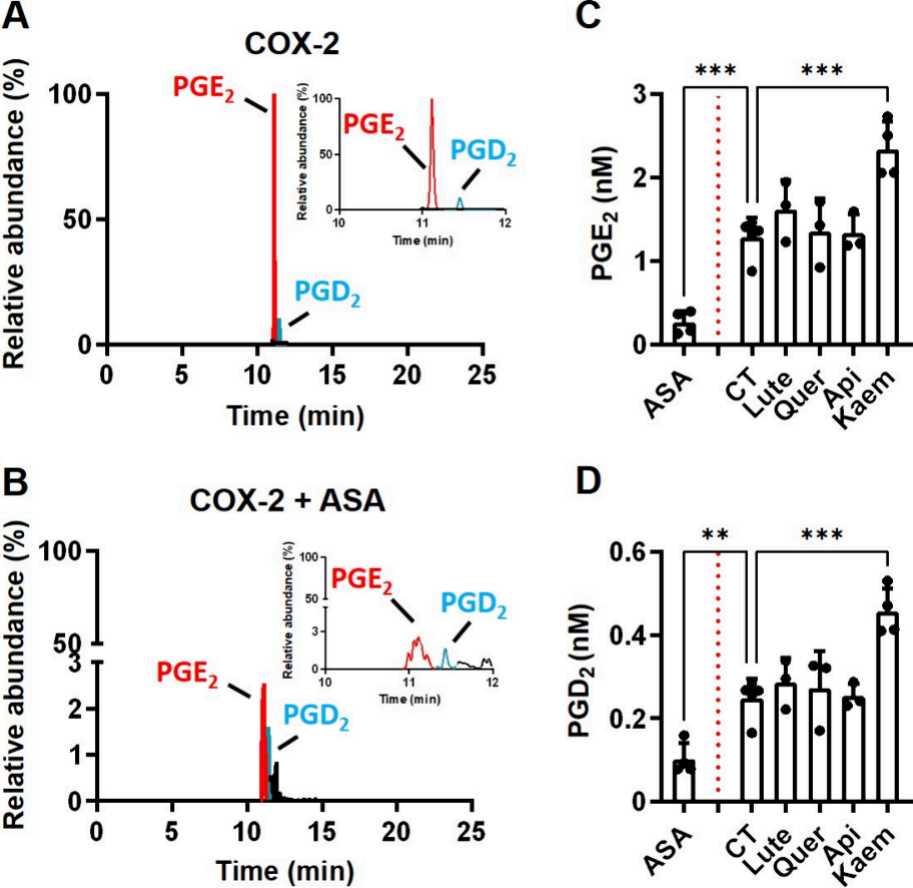
Effect of BW flavonoids (Lute, Quer, Api, and Kaem) on the *in vitro* enzymatic activity of COX-2. Representative chromatograms of hCOX-2 reactions in the absence (A) or in the presence of 1 mM ASA (B). The effect of the BW flavonoids (Lute, Quer, Api, and Kaem) on PGE_2_ (C) and PGD_2_ (D) formation was tested via incubation of the reaction mixture with 15 μM BW flavonoids for 1 h, followed by the addition of 30 μM AA for 15 min in 100 μL buffer (100 mM NH_4_OAc, pH 8) at room temperature. The results are shown as mean ± SD from independent incubations (*n* = 3–4). ANOVA analyses followed by the Holm–Sidak *post hoc* test were used to determine statistically significant differences: **p < 0.01 and *p* < 0.001 versus the control reaction.

**Figure 8:**
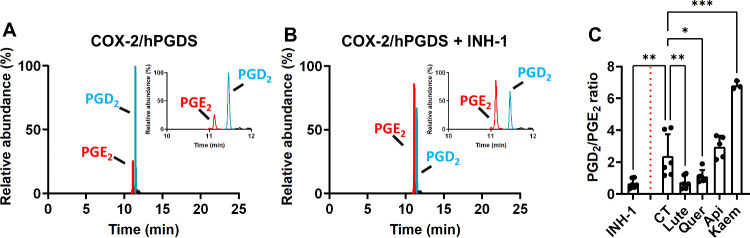
Effect of BW flavonoids (Lute, Quer, Api, and Kaem) on the *in vitro* enzymatic activity of the COX-2/hPGDS tandem. Illustrative chromatograms of the hCOX-2/hPGDS reaction in the absence (A) or the presence of 10 μM INH-1 (B). The effect of the BW flavonoids on the PGD_2_/PGE_2_ (C) was tested by incubating the reaction mixture with 15 μM BW flavonoids for 1 h, followed by the addition of 30 μM AA for 15 min in 100 μL of buffer (100 mM NH_4_OAc, pH = 8) at room temperature. The reaction mixture was extracted as described and analyzed using the UPLC-QTOF. The results are shown as mean ± SD from independent incubations (*n* = 3–6). ANOVA analyses followed by the Holm–Sidak *post hoc* test were used to determine statistically significant differences: *p < 0.05, **p < 0.01, and ***p < 0.001 versus control reaction.

### 3.5 Reducing, Antioxidant, and Chelating Activities of BW Phenolic Acids and Flavonoids

In general, the reducing power of the phenolic acids (0.27–0.43 mM) was lower compared with that observed for flavonoids (0.32–0.90 mM), as shown in Table 2and Table S3. Focusing on those flavonoids that modulate PG synthesis (Figure 2), Quer was the compound that showed the highest reducing activity (and the lowest anodic peak potential), followed by Kaem, Lute, and Api (Table 2). Because BW flavonoids can react with oxidant species via various mechanisms, we performed additional spectrophotometric analyses to assess their antioxidant, reducing (FRAP assay), and chelating activity. As described in Supplementary results (Supporting Information), BW flavonoids showed higher antioxidant, reducing, and chelating activity than phenolic acids (Table S5). Within the flavonoid group, our results showed discrepancies in the antioxidant (DPPH assay), reducing (FRAP experiment), and chelating (FZ test) activities of Quer, Lute, and Kaem compared with Api. The low antioxidant/reducing and high chelating activities of Api were at odds with the effects exerted by Quer (the highest antioxidant/reducing and lower chelating activities), Lute, and Kaem (Table 2).

**Table 2:** Reducing, Antioxidant, and Chelating Activities of BW Flavonoids Provided by CV and Spectrophotometric Assays^a^

	reducing activity			chelating activity	antioxidant activity
	CV		FRAP	FZ	DPPH RSA
compound/assay	anodic peak potentials *E*_p,a_ [V]	Trolox equivalent (mM)		percentage (%)	Trolox equivalent (mM)
Lute*	0.40 ± 0.02^c^	0.57 ± 0.02^d^	1.40 ± 0.03^e^	70.94 ± 0.15^d^	2.07 ± 0.05^a^
Quer*	0.33 ± 0.01^c^	0.90 ± 0.07^a^	2.58 ± 0.03^a^	68.96 ± 0.33^f^	2.09 ± 0.03^a^
Api*	0.87 ± 0.08^b^	0.35 ± 0.01^f^	0.02 ± 0.01^g^	80.29 ± 0.12^a^	0.11 ± 0.02^f^
Kaem*	0.40 ± 0.01^c^	0.74 ± 0.02^b^	1.89 ± 0.05^b^	76.08 ± 0.11^b^	1.17 ± 0.03^e^

a Results provided by the CV, FRAP—ferric-reducing/antioxidant power assay, FZ—ferrozine assay, and DPPH RSA—DPPH radical scavenging activity assay. Data are expressed as means ± SD (*n* = 6). Means in a column related to a respective assay, followed by the different letters, are significantly different (*p* < 0.05) based on the one-way analysis of variance (ANOVA). *The concentrations used were 500 μM for the CV assay and 1 mM for the FRAP, FZ, and DPPH experiment.

### 3.6 Lute, Quer, and Kaem Undergo HRP/H_2_O_2_-Catalyzed Transformation

To explore whether the oxidation of BW flavonoids results in the transformation of the original molecule into an oxidized derived metabolite, we monitored the changes in the UV/vis spectra. Thus, we recorded the spectra of 50 μM Lute, Quer, Api, and Kaem (diluted in 1 mL of NH_4_OAc buffer containing 200 μM H_2_O_2_) before (initial time point; red line) and after the addition of HRP. Lute, Quer, and Kaem underwent HRP-catalyzed oxidative transformation, evidenced by changes in their UV/vis spectra (which could result in the formation of oxidized metabolites), whereas the Api spectrum remained stable throughout the reaction time (Figure 9).

**Figure 9:**
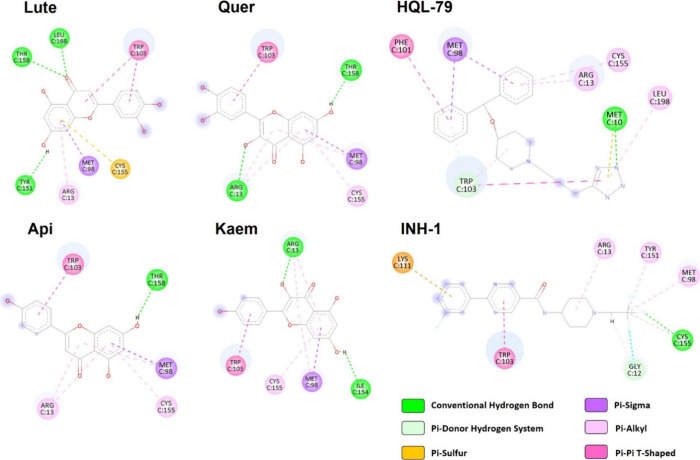
Schematic depiction of the 2D structure of the binding hotspots of BW flavonoids to hPGDS. Different colors represent the types of interactions established between the compounds and the key residues that regulate enzymatic activity.

### 3.7 BW Flavonoids Reduce the Biosynthesis of TNF-α in LPS-Stimulated RAW 264.7 Macrophages

Because LPS increases TNF-α biosynthesis,^45^ and this pro-inflammatory cytokine modulates COX-2 expression in macrophages,^46^ we also investigated the effect of BW flavonoids on TNF-α biosynthesis as an additional mechanism to explain their effects on the LPS/COX-2 route. RAW 264.7 macrophages incubated with 100 ng/mL of LPS induced the biosynthesis of TNF-α. The presence of 15 μM Lute, Quer, Api, and Kaem reduced the TNF-α levels to a similar extent (Figure 5).

### 3.8 BW Flavonoids Modulate the COX-2/hPGDS Enzymatic Reaction

PGs inhibition might also be achieved through direct inhibition of the COX-2 and hPGDS activity. To explore this possibility, we first tested the former and incubated recombinant purified hCOX-2 with 1 mM ASA (inhibition control)^47^ or 15 μM BW flavonoids (Lut, Quer, Api, and Kaem) for 1 h prior to testing their effect on the conversion of AA to PGE_2_ and PGD_2_ using UHPLC-QTOF-MS.

Incubation of hCOX-2 with 30 μM AA for 15 min gave PGE_2_ as the major product, while PGD_2_ was detected at a lower concentration (a PGE_2_/PGD_2_ ratio of 4.5/1; Figure 6A). The same incubation in the presence of 1 mM ASA resulted, as anticipated, in an inhibition of the formation of both PGs (Figure 6B). This inhibitory effect was absent in the presence of BW flavonoids at 15 μM. PGE_2_ and PGD_2_ formation remained stable in the presence of Lute, Quer, and Api. In contrast, Kaem promoted their synthesis, indicating that their inhibitory effect on PG formation is independent of the modulation of COX-2 (Figure 6C,D).

We therefore tested whether hPGDS might be a target of the BW flavonoids to explain the reduced PGD_2_ level observed in LPS-stimulated RAW 264.7 cells. The analysis of the COX-2/hPGDS tandem reaction showed a less efficient formation of PGE_2_ than of PGD_2_ (ratio 1/6.6; Figure 7A). The hPGDS inhibitor-1 (INH-1) shifted this ratio to 1.4/1, favoring PGE_2_ production (Figure 7B). Lute and Quer, at 15 μM, had an effect similar to that of the inhibitor, increasing the enzymatic synthesis of PGE_2_ and consistently reducing PGD_2_. Kaem, at the same concentration, exerted the opposite effect, increasing the PGD_2_/PGE_2_ ratio, whereas Api lacked the capacity to interfere with the tandem reaction (Figure 7C).

### 3.9 *In Silico* Docking Studies on TLR4, COX-2, and hPGDS

Docking simulations were performed on three inflammation-related targets, i.e., hPGDS, Toll-like receptor 4/MD-2 (TLR4/MD-2), and COX-2. The reference ligands (flavonoids and recognized inhibitors) reproduced the expected binding modes, supporting the reliability of the docking protocol. Flavonoids consistently showed favorable predicted binding energies (Table S2) and interaction profiles (Figure 8 and Figures S4 and S5), suggesting their potential role as multitarget anti-inflammatory modulators.

At the TLR4/MD-2 complex, the reference antagonist eritoran docked into the hydrophobic pocket of MD-2 (−4.6 kcal/mol), mainly through hydrophobic and aromatic interactions. Among flavonoids, Api (−7.2 kcal/mol) and Lute (−7.1 kcal/mol) established π–π stacking with Tyr131 or Phe151, π–σ interactions with Ile52 or Ile153, and multiple hydrophobic contacts with Ile32, Ile124, and Ile154. Quer and Kaem (both −6.8 kcal/mol) interacted mainly through π–σ with Ile153 and π–alkyl/hydrophobic contacts with Ile32, Ile52, Ile61, and Val48 (Table S2 and Figure S4).

For COX-2 (Table S2 and Figure S5), the selective inhibitor celecoxib displayed the strongest affinity (−12.1 kcal/mol). Flavonoids exhibited consistent affinities ranging from −9.2 kcal/mol (Kaem, Quer, and Lute) to −9.0 kcal/mol (Api). Kaem and Quer interacted through π–σ with Val492 and Leu321, hydrogen bonds with Gln161, Tyr324, and Phe487, and hydrophobic contacts with Val318 and Ala485. Lute displayed a comparable profile, with an additional hydrogen bond to Ser499, although it also showed an unfavorable donor–donor interaction with Phe487. Api engaged in π–σ interactions with Val318, Val492, and Ala496, along with π–alkyl contacts with Leu321 (Figure S5).

Importantly, the most pronounced and homogeneous binding profiles were observed for hPGDS. The catalytic pocket of hPGDS (Trp103/Arg13 in Mcule numbering) accommodated the reference inhibitors HQL-79 and INH-1 with predicted binding affinities of −7.8 and −7.9 kcal/mol, respectively (Table S2). All flavonoids tested exhibited stronger affinities than HQL-79, both inhibitors, ranging from −8.6 kcal/mol for Kaem to −8.0 kcal/mol for Lute. Their binding poses reproduced the interactions of the inhibitors: π–π stacking with Trp103, hydrogen bonding or π–alkyl interactions with Arg13, and additional stabilizing contacts with Met98, Cys155, Ile154, Thr158, Leu198, and Tyr151 (Figure 8). These conserved binding patterns suggest that flavonoids could act as competitive inhibitors of hPGDS, potentially interfering with the PGD_2_ biosynthesis.

## 4 Discussion

A fundamental feature of BW-derived products is their chemical complexity, which is particularly relevant in relation to the variability of their (poly)­phenolic composition. Rutin, a representative dietary molecule in BW, exemplifies this complexity since its concentration differs markedly across varieties or preparations. This variability is also inherent to other flavonoids and phenolic acids present in BW,^48^ leading to ambiguity about the biological effects of individual (poly)­phenols and the benefits associated with the intake of different BW products.

Information on specific bioactive components (mechanisms of action, cellular targets, and structure–activity relationships) is critical to identify the indispensable molecules responsible for the effects of interest (e.g., anti-inflammatory effects). Against this background, herein, we examined the capacity of a range of flavonoids and phenolic acids present in BW (Figure 1) to target the LPS/COX-2 pathway in RAW 264.7 macrophages.

Macrophages are essential components of the immune system that can be a double-edged sword at the intestinal level. While they act as peacekeepers in maintaining intestinal homeostasis, activated macrophages can become drivers of intestinal inflammation.^49^ LPS stimulation of macrophages is a well-recognized inflammatory trigger, resulting in TLR4 activation and stimulation of cytokines (i.e., TNF-α) and COX-2-derived PGs biosynthesis.^49–51^ PGE_2_ and PGD_2_ are widely investigated molecules that are considered important biomarkers of intestinal inflammation. Both PGs show elevated levels at the inflammation sites, disrupting the pro-/anti-inflammatory equilibrium, which, in turn, promotes intestinal inflammation.^52^ Nonetheless, reducing high PGE_2_ levels using natural compounds is a commonly used strategy to attenuate inflammation. In contrast, the study of the effects on PGD_2_ formation has been much less approached. The “targeted eicosanoid” methodology used in this study enabled us to analyze both PGE_2_ and PGD_2_, providing a broader perspective (e.g., compared with ELISA assays) on the effects of the (poly)­phenols tested on their biosynthesis.

Among the range of BW phenolic acids and flavonoids evaluated (Figure 1), we identified PGE_2_ and PGD_2_ as targets of Lute, Api, Quer, and Kaem at *in vivo* relevant concentrations^27,28^ (from 1 to 15 μM; Figure 2). The effect (or lack of it) of these four compounds on PGE_2_ is comparable to that previously reported at similar concentrations and the same cellular assay.^53,54^ In contrast, their effect on PGD_2_, as far as we are aware, is unprecedented in this *in vitro* model. The other compounds evaluated were ineffective in modulating PG biosynthesis (Figure S2). Their inactivity on PGE_2_ biosynthesis is in accordance with^55–57^ or in contrast to^58–60^ *in vitro* studies using the same cellular model but different experimental conditions. Factors such as LPS dose, time of treatment, (poly)­phenol concentration used, and/or adjustment of the FBS proportion to avoid interference with COX-2 expression^29^ are factors to consider in explaining the differences observed.

Metabolic transformation of the compounds investigated is another important aspect to consider in explaining differences in their bioactivity.^61^ In addition to the described capacity of RAW 264.7 macrophages to conjugate with glucuronic acid,^62^ our results further showed, for the first time, that these cells can form sulfates, methyl ester metabolites, or combinations of the two (Table 1). This intricate metabolic model resulting from the tandem interaction of different phase-II metabolizing enzymes offers interesting insights into the structural modifications of (poly)­phenols and their influence on bioactivity. In our study, however, it is unclear whether these metabolic reactions are related to the (in)­activity exhibited by the dietary compounds tested. One reason is the absence of a direct relationship between a compound’s tendency to undergo phase-II metabolism and their bioactivity. For example, phenolic acids were inactive regardless of whether they remained in their original form (e.g., caffeic acid) or acted as precursors of derived metabolites (e.g., vanillic acid), whereas the majority of flavonoids underwent phase-II metabolism, yet not all were active (e.g., Lute vs epicatechin). Another reason is the absence of studies about the biological activity of the phase-II metabolites formed (e.g., methyl Lute glucuronide); let alone the mixture of metabolites as detected in our assay. This raises the question of whether the flavonoids (before being metabolized) or their derived metabolites might act as active principle(s) able to target macrophages regarding inflammation modulation.

To integrate these observations, a mechanistic understanding of the inhibitory effects of Lute, Api, Quer, and Kaem on PGE_2_ and PGD_2_ biosynthesis requires the investigation of relevant upstream branch points. In LPS-treated RAW 264.7 macrophages, PGs arise from the oxidation of arachidonic acid, mainly by COX-2, to form PGH_2_ that undergoes further conversion into PGD_2_ as the predominant product and PGE_2_ as a minor compound.^51^ Given the pivotal role of COX-2 in PG formation, the first mechanism explored was regulation of the COX-2 protein levels. According to the targeted eicosanoid assay, Lute, Api, and Kaem reduced COX-2 levels (Figure 3), an effect consistent with prior studies showing that these compounds downregulate COX-2.^53,54^ However, the significant increase observed in the presence of Quer (Figure 3) conflicts with the PGs level detected in the culture media. Studies using the same cellular model and similar assay conditions with Quer reported a comparable pattern, characterized by increased COX-2 levels while sparing PGE_2_ production.^53,63^ At this point, we explored further mechanisms to find possible explanations for these contrasting effects.

TLR4/MD-2 receptor constitutes a pivotal upstream point in the modulation of the LPS-triggered reaction in macrophages. The *in silico* simulation revealed a profile of targeted residues unrecognized to date (Figure S4), which refines and complements the results described in previous studies.^64–68^ This prediction reinforces the role of the TLR4/MD-2 complex as an important binding partner of BW flavonoids, although whether this interaction leads to inhibitory effects requires additional experimentation. Lute and Kaem inhibited the phosphorylation of Ser_177_ and Ser_181_ of IKKβ (Figure 4), a critical step in the LPS-triggered activation of NF-κB and related downstream pathways, including COX-2 expression. A likely associated mechanism might involve adduction of Cys-179-IKKβ by these compounds and/or their oxidation metabolites.^69,70^ Their higher antioxidant/radical scavenging activity, along with their susceptibility to undergo oxidative transformations (as reflected in Figure 5), compared to Api (inactive compound), supports this postulation. Nevertheless, the absence of an effect exerted by Api and Quer, unanticipated considering their effects on COX-2, suggests the implication of additional mechanisms beyond their antioxidant capacity and possible oxidative transformations. A different way to approach is the modulation of relevant molecules, such as TNF-α, involved in the activation of the NF-κB pathway.^45^ TNF-α modulation partially explains the effect of the pathway since the four flavonoids exerted a significant reduction of the cytokine regardless of the effect observed on COX-2 (Figure 6).

An alternative explanation resides in the modulation of the catalytic activity of the enzymes involved in PG synthesis. COX-2 catalyzes a series of oxygenation steps that involve free radical reactions to convert AA into PGG_2_, which is reduced to form the PG precursor known as PGH_2_.^71^ Our docking simulation, we identified relevant amino acid residues in the active site that interact with our compounds, potentially interfering with COX-2 catalytic activity of COX-2 through their radical scavenging properties (Figure S5). This could be a plausible mechanism considering the hampering effects of Lute, Quer, and Kaem (high antioxidant and low chelating activity) on COX-2 catalytic activity^72–74^ and the inability of Api (with an opposite profile) to suppress the reaction.^75^ However, our results indicated that Lute, Api, and Quer lacked an effect on COX-2 activity, whereas Kaem stimulated PG formation (Figure 7). The effect of Api might be anticipated,^75^ and the discrepancies with other studies for the other compounds could be associated with (i) the source of COX-2 (e.g., rh-COX-2 vs rat renal medulla origin;^74^ (ii) the variability of the concentrations tested (our concentration is below the IC_50_ = 100–490 μM reported elsewhere^73,74,72^ ) is a relevant factor considering the hormetic behavior of our compounds;^72–77^ and (iii) the analytical methods used for PG production.^73,74,72^

We next investigated their effects on hPGDS to understand their role in PG biosynthesis, as we were unable to explain the effects of our compounds via the COX-2 inhibition. hPGDS is an enzyme constitutively expressed in RAW 264.7 macrophages that catalyzes the conversion of the COX-2-derived PGH_2_ into PGD_2_,^78,79^ which is the predominant PG formed in this cellular model. Research exploring the influence of natural products on hPGDS activity as an anti-inflammatory mechanism is sparse. Alkaloid natural extracts^80^ and tannic acid^81^ act as inhibitors of the hPGDS catalytic activity in cell-free biochemical assays. We tested the effect of our compounds using the COX-2/hPGDS biochemical model to mimic, to the extent possible, the RAW 264.7 cells machinery. Lute and Quer reduced the PGD_2_/PGE_2_ ratio to a level comparable to that achieved by the specific hPGDS inhibitor. In contrast, Api and Kaem were ineffective as inhibitors, as the ratio remained unaltered or increased roughly 20-fold, respectively, compared to that of the control reaction (Figure 8). Given the lack of comparable studies, we conducted additional *in silico* analyses to explain the dissimilar effects observed. Binding affinities obtained from docking simulations were insufficient to explain the discrepancies observed on hPGDS, since the predicted values for reference inhibitors and BW flavonoids were similar. However, the differences in the binding patterns of the compounds depicted in Figure 9 may help clarify the dissimilar biological activities described. Thus, the interaction between Kaem and Ile154 could modify the activity of the adjacent Cys155, a residue implicated in the regulation of PGDS activity,^82^ thereby increasing enzyme activity. The inhibition of hPGDS by Lute may stem from binding to Tyr151 and Leu198, key residues involved in stabilizing the recognized inhibitors.^82^ Quer and Api bound the same residues, although their interaction with Arg13 (hydrogen bond and pi-alkyl, respectively) was different, which may affect their disposition in the active site and, therefore, explain their contrasting effects on the enzymatic activity. As described elsewhere,^83^ experiments using hPGDS mutants for the predicted interacting amino acids are critical to confirm their hypothetical role in the ligand–receptor interaction and their implication in biological activity.

In this study, we investigated specific mechanisms (some unexplored to date) tailored to the relevant characteristics of the RAW 264.7 macrophages. Beyond the commonly assessed markers (PGE_2_ and COX-2 protein levels), we demonstrated that PGD_2_ (the major compound formed), hPGDS (highly expressed in this model), and COX-2 enzymatic activity or Ikkβ also act as targets of BW flavonoids, exerting their therapeutic mechanisms at different nodes of the LPS/COX-2 pathway. This helps unravel paradoxical effects, making it easier to interpret the observed effects observed. For instance, Quer reduces PGD_2_ biosynthesis, despite increasing COX-2 levels by inhibiting hPGDS activity. Even though these results might serve as a starting point for new research, several limitations deserve attention. The relevance of results from cell-free experiments (e.g., enzymatic incubations) for explaining effects in cellular assays (e.g., LPS-activated macrophages) is limited. The potency of BW flavonoids in intact cells can be different from that observed in cell-free *in vitro* reaction assays. Moreover, *in silico* docking analyses are useful for predicting ligand–target interactions (e.g., between BW flavonoids and TLR4), but are still insufficient to infer biological activity. Additional experimental approaches (e.g., receptor-binding assays) are required to validate the predicted interactions. Identifying active principles in BW and elucidating mechanisms of action provide important scientific evidence on how diet contributes to disease prevention (e.g., via targeting inflammation). This also supports maintaining good health and well-being (i.e., Sustainable Development Goal 3; SGD3). It could also help obtain BW products enriched in these bioactive compounds (e.g., through genomics and transcriptomics approaches), thereby enabling the preparation of more sophisticated BW-derived functional foods that align with consumer demands and improve the quality of life of the population.

## Supplementary Material



## Notes

Supplementary methods: cell viability assay, handling conditions, metabolite extraction, and Ikkβ phosphorylation; supplementary results: cell viability effects, study of the effect of BW phenolic acids and flavonoids, and time-course phosphorylation; and SI figures and tables (PDF)
