# Supplementary material for: Buckwheat Flavonoids Modulate Inflammation in RAW 264.7 Macrophages at Physiologically Relevant Concentrations via the LPS/COX‑2 Pathway
Source: J Agric Food Chem. 2026 Mar 13;74(11):9441–54. doi: 10.1021/acs.jafc.6c02109 (PMC13022854; doi:10.1021/acs.jafc.6c02109)
Supplement: Supplementary file 1 [file jf6c02109_si_001.pdf]

Supporting Information

Buckwheat flavonoids modulate inflammation in RAW 264.7 macrophages at physiologically relevant concentrations via the LPS/COX-2 pathway

Diego José López-Cánovas[a], Antonio Vico-Padilla[a,b], Danuta Zielińska[c], Sabrina Poveda-Lora[a], Silvia Navarro-Orcajada[a], David López-Martínez[d], Diana García-Moreno[e,f], María Ángeles Ávila-Gálvez[a,b], Beatriz Garay-Mayol[a], José E. Yuste[g], Fernando Vallejo[g], Juan Carlos Espín[a,b], Antonio González-Sarrías[a], Henryk Zielinski[h,]* and Juan Antonio Giménez-Bastida[a,b,]*

[a]Quality, Safety & Bioactivity Plant Foods, Food Science & Technology Dep., CEBAS-CSIC, 30100 Murcia, Spain

[b]Food and Health Laboratory, Food Science and Technology Dep., CEBAS-CSIC, Murcia 30100, Spain

[c]Department of Chemistry, University of Warmia and Mazury, Olsztyn 10-721, Poland

[d]Dep. Biochemistry and Molecular Biology-A, Faculty of Veterinary, University of Murcia, Murcia 30100, Spain

[e]Center for Biomedical Research in Rare Diseases Network (CIBERER), Carlos III Health Institute, Madrid 28029, Spain

[f]Biomedical Research Institute of Murcia (IMIB)-Pascual Parrilla, Murcia 30120, Spain

[g]Metabolomics Platform, CEBAS-CSIC, Murcia 30100, Spain

[h]InLife Institute of Animal Reproduction and Food Research, Team of Chemistry and Biodynamics of Food, Polish Academy of Science, Władysława Trylińskiego 18, Olsztyn 10-683, Poland

Co-corresponding authors:

*Juan A. Giménez Bastida, Ph.D., email: jgbastida@cebas.csic.es

*Henryk Zielinski, Professor, Professor, email: h.zielinski@pan.olsztyn.pl

Table of Contents

Supplementary methods...Page 3

Supplementary results ....Page 5

References..Page 23

SUPPLEMENTARY METHODS

Cell viability assay. The effects of BW phenolic acids and flavonoids on cell viability were tested at the highest concentration used (15 μM) and measured by MTT reduction[1]. Confluent cells (≥90% confluence) seeded in 96-well plates were incubated in fetal bovine serum (FBS)-free medium for 24 h. Next, we treated the macrophages with 10 μg/mL LPS alone or in the presence of phenolic acids and flavonoids at 15 μM (DMSO ≤ 0.5% v/v) for 4 h. Under the same conditions, untreated cells (DMSO ≤ 0.5% v/v) were used as a control, and their viability was set at 100% for comparison. The culture medium was removed, the cells washed with PBS and incubated at 37 °C in sterile-filtered red phenol-free culture medium containing 1 mg/mL MTT for 45 min. The MTT-supplemented medium was removed, the formazan crystals were solubilized in 100 μL DMSO, and absorbance was measured at 570 and 690 nm (test and reference wavelength, respectively) using a Fluostar Galaxy spectrophotometer (BMG Lab. Technologies v5.0). Cell viability was assessed using three assays (n = 3), with each treatment repeated 6 times (6 wells per compound tested).

Handling conditions of RAW 264.7 macrophages and THP-1 monocytes. The RAW 264.7 macrophages were incubated at 37 °C, 7.5% CO_2 and 95% relative humidity. As part of routine handling, we seeded the macrophages at an initial density of 10,000 cells/cm^2 and added fresh complete medium every 2 or 3 days until reaching 90% confluence, after which they were subcultured. Thawed cells (first passage) were subcultured at least two times before the initiation of the cellular experiments, which were performed within passages 4 and 12.

The maintenance and growth conditions of the THP-1 monocytes were 37 °C and 5% CO2/95% air atmosphere. The cellular concentration was maintained between 3×10^5 and 8×10^5 cells/mL, and the passage number was between 15 and 20 to keep the cells in optimal conditions.

Metabolites extraction from the culture medium. We mixed 1 mL culture medium with an equal volume of 0.2% (v/v) acetic acid (final pH = 3.5) containing 10 μM PGE_2–d_4 prior to loading the samples onto preconditioned (activation with 3 mL MeOH and washing with 3 mL H_2O) 100 mg Bond Elut C18 cartridges (Agilent, Santa Clara, CA, USA). After sample loading, we washed the cartridges with water, added 500 μL ethyl acetate to remove excess water, and eluted the retained molecules with 1 mL MeOH. Eluate preparation for analysis involved filtration (0.22 μm cellulose regenerated filters; Agilent), evaporation in a speed-vacuum concentrator (Savant SPD140DDA) and resuspension in 50 μL MeOH.

Ikkβ phosphorylation at Ser177/181 in LPS-treated THP-1 monocytes.

THP-1 monocytes were incubated in FBS-deprived RPMI 1640 medium at 800,000 cells/mL for 1 h before the treatment with 10 μg/mL LPS for 15 and 30 min. Protein extraction and Ikkβ phosphorylation at Ser177/181 was performed according to the manufacturer's instructions for PathScan® Phospho-Ikkβ sandwich ELISA kit (#7080) from Cell Signaling Technology (MA, USA). This kit was obtained from Werfen (Barcelona, Spain) as the official distributor.

SUPPLEMENTARY RESULTS

Cell viability effects.

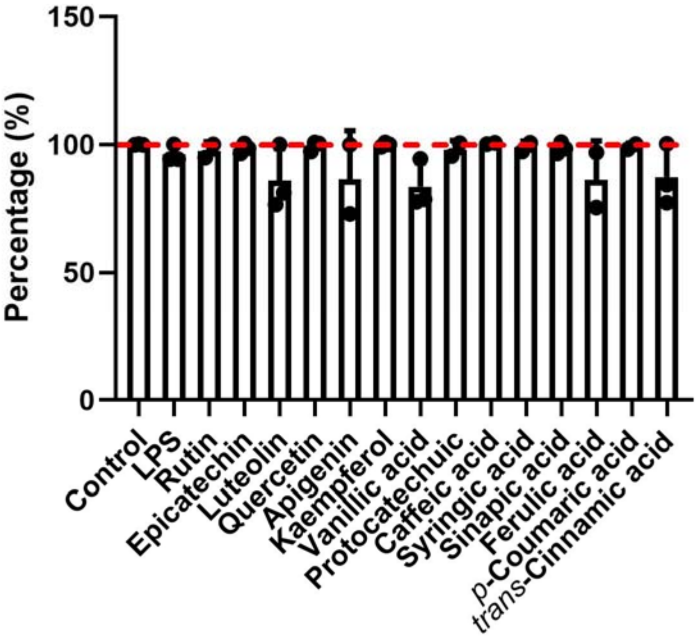

Figure S1. Evaluation of the cytotoxic effect of BW phenolic acids and flavonoids (15 μM) on LPS-activated RAW 264.7 macrophages. The results show the average ± SD of viable cells compared to untreated macrophages (set as 100% cell viability). The results come from three independent experiments (n=3), in which each treatment was repeated six times (6 wells per treatment).

Study of the effect of BW phenolic acids and flavonoids on PGs biosynthesis.

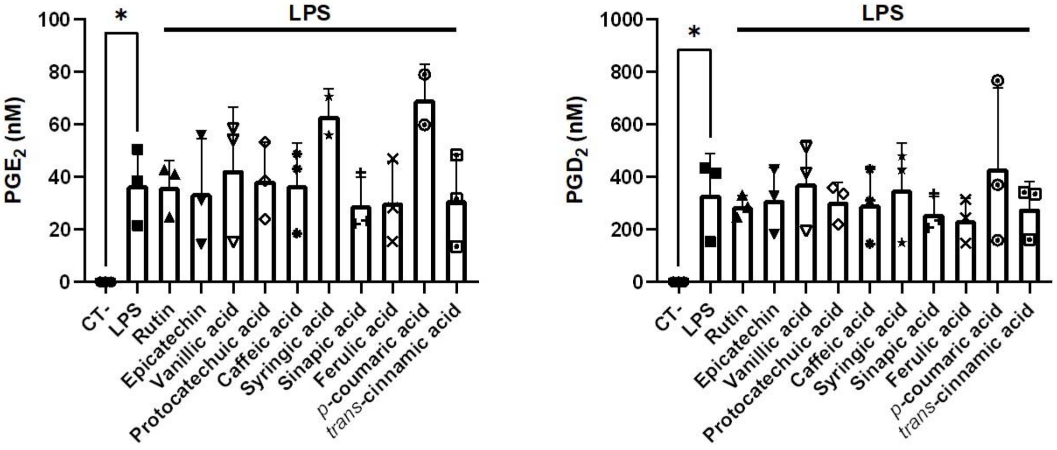

Figure S2. Determination of the level of PGE_2 and PGD_2 in culture medium of LPS-activated RAW 264.7 macrophages in the presence of 15 μM BW phenolic acids and flavonoids. The bar graphs, displayed as mean ± SD, illustrate the results obtained from 3 independent experiments (n = 3). ANOVA analysis and Bonferroni *post hoc* test were used to determine statistically significant differences: *, $p<0.05$; ***, $p<0.001$ *versus* LPS-stimulated RAW 264.7 cells.

Time-course phosphorylation of Ikkβ in LPS-treated THP-1 monocytes at 15 and 30 min.

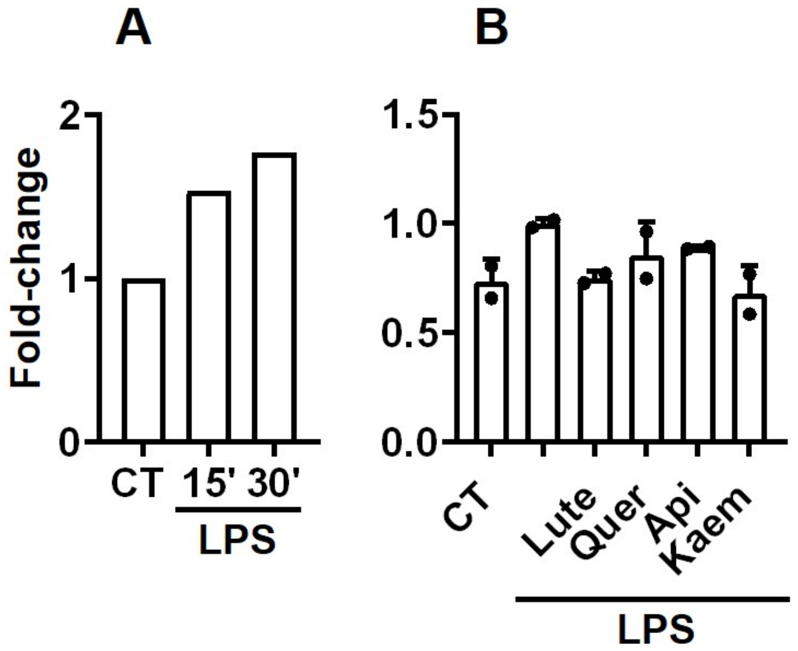

Figure S3. Induction of Ikkβ phosphorylation at Ser177/181 in THP-1 monocytes. (A) Time-course study of the Ikkβ phosphorylation in THP-1 monocytes treated with 10 μg/mL LPS for 15 and 30 min (n=1). (B) Screening of the inhibitory effect of BW flavonoids on the Ikkβ phosphorylation (n=2). Data are shown as mean ± SD.

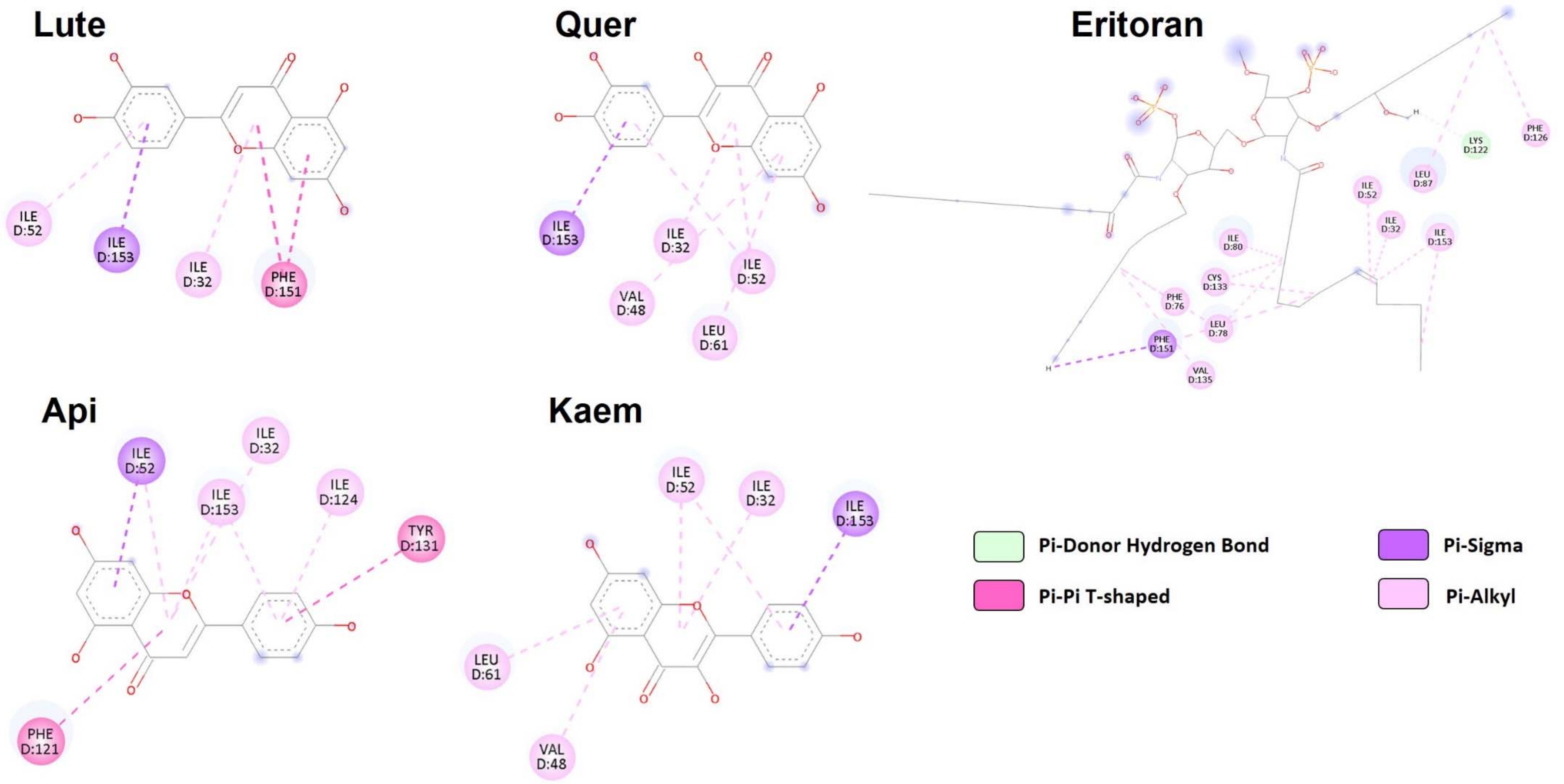

Figure S4. Schematic depiction of the theoretical interactions between the BW flavonoids or reference inhibitors (eritoran) and the enzymatic residues of the complex TLR4-MD.

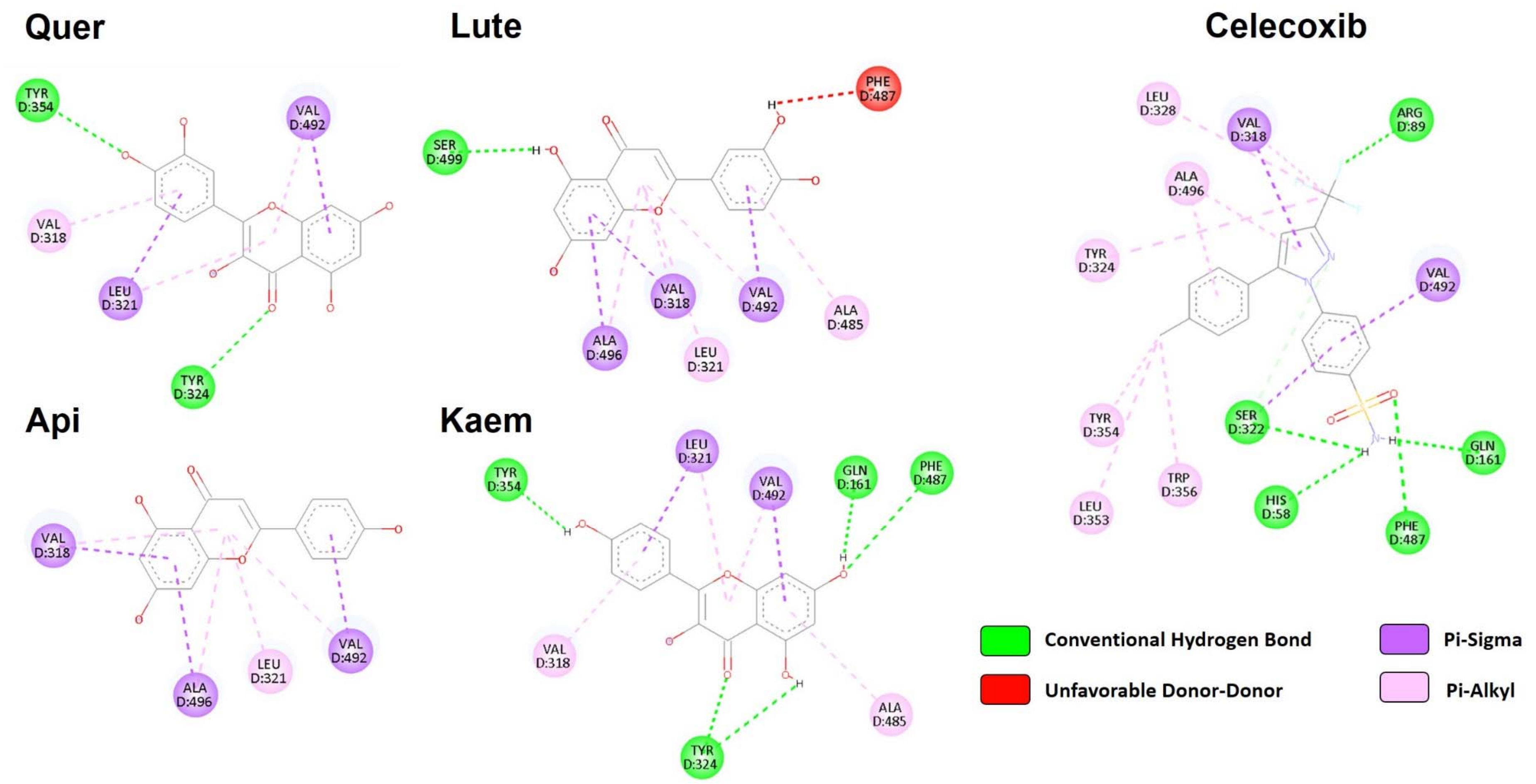

Figure S5. Schematic depiction of the theoretical interactions between the BW flavonoids or reference inhibitors (celecoxib) and key residues of COX-2 involved in the regulation of the enzymatic activity.

Table S1. Molecular formulas and theoretical *m/z*⁻ values of BW phenolic acids and flavonoids, and their phase II metabolites, identified after *in vitro* incubations with RAW 264.7 macrophages.

	Formula	m/z^-
**Rutin treatment**		
**Rutin**	$C_{27}H_{30}O_{16}$	609.1461
Quercetin	$C_{15}H_{10}O_7$	301.0354
Quercetin glucuronide	$C_{21}H_{18}O_{13}$	477.0675
**Epicatechin treatment**		
**Epicatechin**	$C_{15}H_{14}O_6$	289.0718
Epicatechin glucuronide	$C_{15}H_{22}O_{12}$	393.1038
Epicatechin diglucuronide	$C_{21}H_{30}O_{18}$	569.1359
Epicatechin sulfate	$C_{15}H_{14}O_9S$	369.0286
Methyl epicatechin	$C_{16}H_{16}O_6$	303.0874
Methyl epicatechin glucuronide	$C_{22}H_{24}O_{12}$	479.1195
Methyl epicatechin sulfate	$C_{16}H_{16}O_9S$	383.0442
**Luteolin treatment**		
**Luteolin**	$C_{15}H_{10}O_6$	287.0550
Luteolin glucuronide	$C_{21}H_{18}O_{12}$	462.0798
Luteolin diglucuronide	$C_{27}H_{26}O_{18}$	637.1046
Luteolin sulfate	$C_{15}H_{10}O_9S$	364.9973
Methyl luteolin	$C_{16}H_{12}O_6$	299.0561
Methyl luteolin glucuronide	$C_{22}H_{20}O_{12}$	475.0882
Methyl luteolin sulfate	$C_{16}H_{12}O_9S$	379.0129
**Quercetin treatment**		
**Quercetin**	$C_{15}H_{10}O_7$	301.0354

Quercetin glucuronide	$C_{21}H_{18}O_{13}$	477.0675
Quercetin diglucuronide	$C_{27} H_{26} O_{19}$	653.0996
Methyl quercetin glucuronide	$C_{22}H_{20}O_{13}$	491.0831
Methyl quercetin	$C_{16}H_{12}O_7$	315.0510
**Apigenin treatment**		
**Apigenin**	$C_{15}H_{10}O_5$	269.0455
Apigenin glucuronide	$C_{21}H_{18}O_{11}$	445.0776
Apigenin diglucuronide	$C_{27}H_{26}O_{17}$	621.1097
Apigenin sulfate	$C_{15}H_{10}O_8S$	349.0024
Methyl apigenin	$C_{16}H_{12}O_5$	283.0612
Methyl apigenin glucuronide	$C_{22}H_{20}O_{11}$	459.0933
Methyl apigenin sulfate	$C_{16}H_{12}O_8S$	363.0180
**Kaempferol treatment**		
**Kaempferol**	$C_{15}H_{10}O_6$	285.0405
Kaempferol glucuronide	$C_{21}H_{18}O_{12}$	461.0725
Kaempferol diglucuronide	$C_{27}H_{26}O_{18}$	637.1046
Kaempferol sulfate	$C_{15}H_{10}O_9S$	364.9973
Methyl kaempferol	$C_{16} H_{12} O_6$	299.0561
Methyl kaempferol glucuronide	$C_{22}H_{20}O_{12}$	475.0882
Methyl kaempferol sulfate	$C_{16}H_{12}O_8$	331.0459
**Vanillic acid treatment**		
**Vanillic acid**	$C_8H_8O_4$	167.0350
Vanillic acid glucuronide	$C_{14}H_{16}O_{10}$	343.0671
Vanillic acid sulfate	$C_8H_8O_7S$	246.9918
Hydroxy vanillic acid	$C_8H_{10}O_5$	185.0455
**Protocatechuic acid treatment**		

**Protocatechuic acid**	$C_7H_6O_4$	153.0193
Protocatechuic acid glucuronide	$C_{13}H_{14}O_{10}$	329.0514
Protocatechuic acid sulfate	$C_7H_6O_7S$	232.9761
**Caffeic acid treatment**		
**Caffeic acid**	$C_9H_8O_4$	179.0350
Caffeic acid glucuronide	$C_{15}H_{16}O_{10}$	355.0671
Caffeic acid sulfate	$C_9H_8O_7S$	258.9918
**Syringic acid treatment**		
**Syringic acid**	$C_9H_{10}O_5$	197.0455
Syringic acid glucuronide	$C_{15}H_{18}O_{11}$	373.0776
Syringic acid sulfate	$C_9H_{10}O_8S$	277.0024
Acetyl syringic acid	$C_{11}H_{12}O_6$	239.0561
Glucosyl syringic acid	$C_{15}H_{20}O_{10}$	359.0984
**Sinapic acid treatment**		
**Sinapic acid**	$C_{11}H_{12}O_5$	223.0612
Sinapic acid glucuronide	$C_{17}H_{20}O_{11}$	399.0933
Sinapic acid sulfate	$C_{11}H_{12}O_8S$	303.0180
**Ferulic acid treatment**		
**Ferulic acid**	$C_{10}H_{10}O_4$	193.0508
Ferulic acid glucuronide	$C_{16}H_{18}O_{10}$	369.0827
Ferulic acid sulfate	$C_{10}H_{10}O_7S$	273.0074
***p*-Coumaric acid treatment**		
***p*-Coumaric acid**	$C_9H_8O_3$	163.0401
p-Coumaric acid glucuronide	$C_{15}H_{16}O_9$	339.0722
p-Coumaric acid sulfate	$C_9H_8O_6S$	242.9969
***trans*-Cinnamic acid treatment**		

***trans*-Cinnamic acid**	$C_9H_8O_2$	147.0452
trans-Cinnamic acid glucuronide	$C_{15}H_{16}O_8$	323.0772
trans-Cinnamic acid sulfate	$C_9H_8O_5S$	227.0020

Table S2. Molecules tested and predicted binding affinities towards the active sites of H-PGDS, TLR4/MD-2, and COX-2 obtained by molecular docking.

**PubChem CID**	**Common name**	**Formula Molecular weight (g/mol)**	**Chemical structure**	**Binding affinity to H-PGDS**	**Binding affinity to TLR4**	**Binding affinity to COX-2**
6540277	HQL-79	$C_{22}H_{27}N_5O$ (377.5)	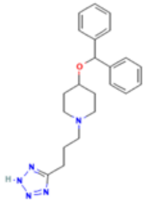	-7.8	-	-
24991044	INH-1	$C_{19}H_{19}F_4N_3O$ (381.4)	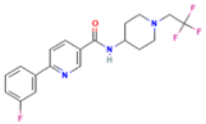	-7.9		

6912404	Eritoran	$C_{66}H_{126}N_2O_{19}P_2$ (1317)	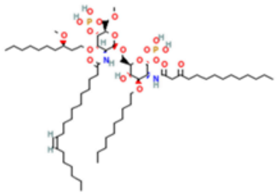	-	-4.6	-
2662	Celecoxib	$C_{17}H_{14}F_3N_3O_2S$ (381.4)	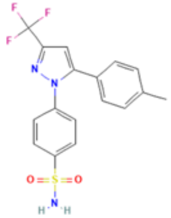	-	-	-12.1
5280343	Quercetin	$C_{15}H_{10}O_7$ (302.23)	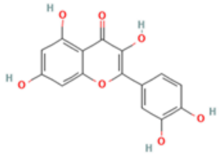	-8.4	-6.8	-9.2
5280863	Kaempferol	$C_{15}H_{10}O_6$ (286.24)	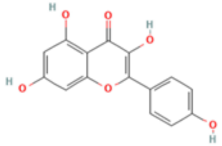	-8.6	-6.8	-9.2
5280445	Luteolin	$C_{15}H_{10}O_6$ (286.24)	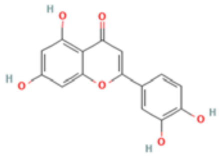	-8.0	-7.1	-9.2

5280443	Apigenin	$C_{15}H_{10}O_5$ (270.24)	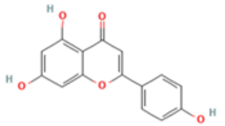	-8.2	-7.2	-9.2

INH-1: Prostaglandin D synthase (hematopoietic-type) inhibitor I

Table S3. The reducing activity of BW phenolic acids and flavonoids (500 μM) as provided by the cyclic voltammetry (CV) method.

Compound/Assay	Anodic peak potentials $E_{p,a}$ [V]	Reducing Activity (mM Trolox)
	Phenolic acids	
Vanillic acid	0.676 ± 0.03[a]	0.27 ± 0.01[b]
Protocatechuic acid	0.465 ± 0.01[c]	0.39 ± 0.02[a]
Caffeic acid	0.334 ± 0.01[c]	0.43 ± 0.05[a]
Syringic acid	0.501 ± 0.02[c]	0.36 ± 0.02[b]
Sinapic acid	0.528 ± 0.02[c]	0.33 ± 0.01[b]
Ferulic acid	0.557 ± 0.07[bc]	0.37 ± 0.01[a]
p-Coumaric acid	0.598 ± 0.04[ab]	0.31 ± 0.02[b]
trans-Cinnamic acid	n.d.	n.d.
	Flavonoids	
Rutin	0.390 ± 0.04[c]	0.46 ± 0.01[e]
Epicatechin	0.339 ± 0.01[c]	0.59 ± 0.05[cd]
Luteolin	0.404 ± 0.02[c]	0.57 ± 0.02[d]
Quercetin	0.334 ± 0.01[c]	0.90 ± 0.07[a]
Apigenin	0.869 ± 0.08[b]	0.35 ± 0.01[f]
Kaempferol	0.398 ± 0.01[c]	0.74 ± 0.02[b]

Data are expressed as means ± SD (n = 6). Means in a column labelled by different letters are significantly different ($p<0.05$) based on the one-way analysis of variance (ANOVA).

Table S4. Cyclic voltammograms of 250 μM of standard solutions of BW phenolic acids (A – H) and flavonoids (I – P) in 0.1 M Britton-Robinson (B-R) buffer pH 6.0 in 80% methanol (v/v) recorded from -0.1 to +1.2 V; scan rate 100 mV s^{-1}. For trolox (Q - reference compound) the cylic voltammograms were analyzed within the range of 0.10 – 2.5 mM in 0.1 M sodium acetate-acetic buffer (pH 4.5) in 80% methanol (v/v) recorded from -100 to +1300 mV; scan rate 100 mV s^{-1}.

PHENOLIC ACIDS

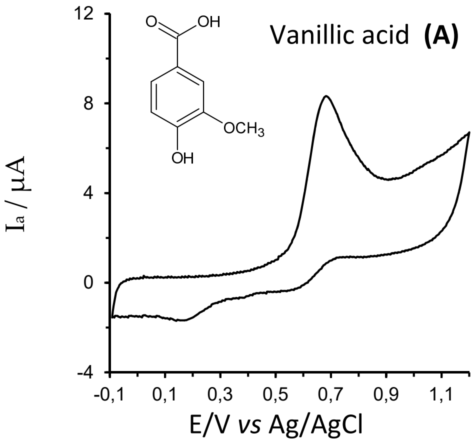

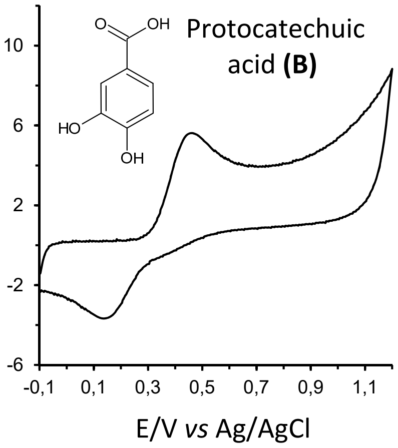

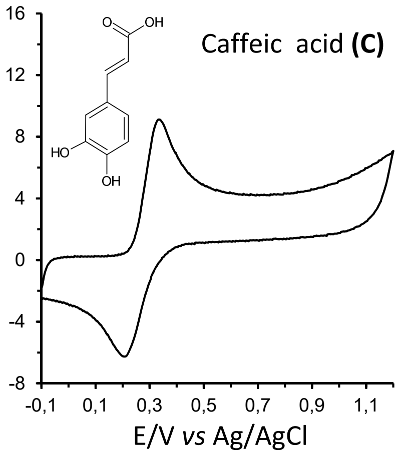

PHENOLIC ACIDS (continued)

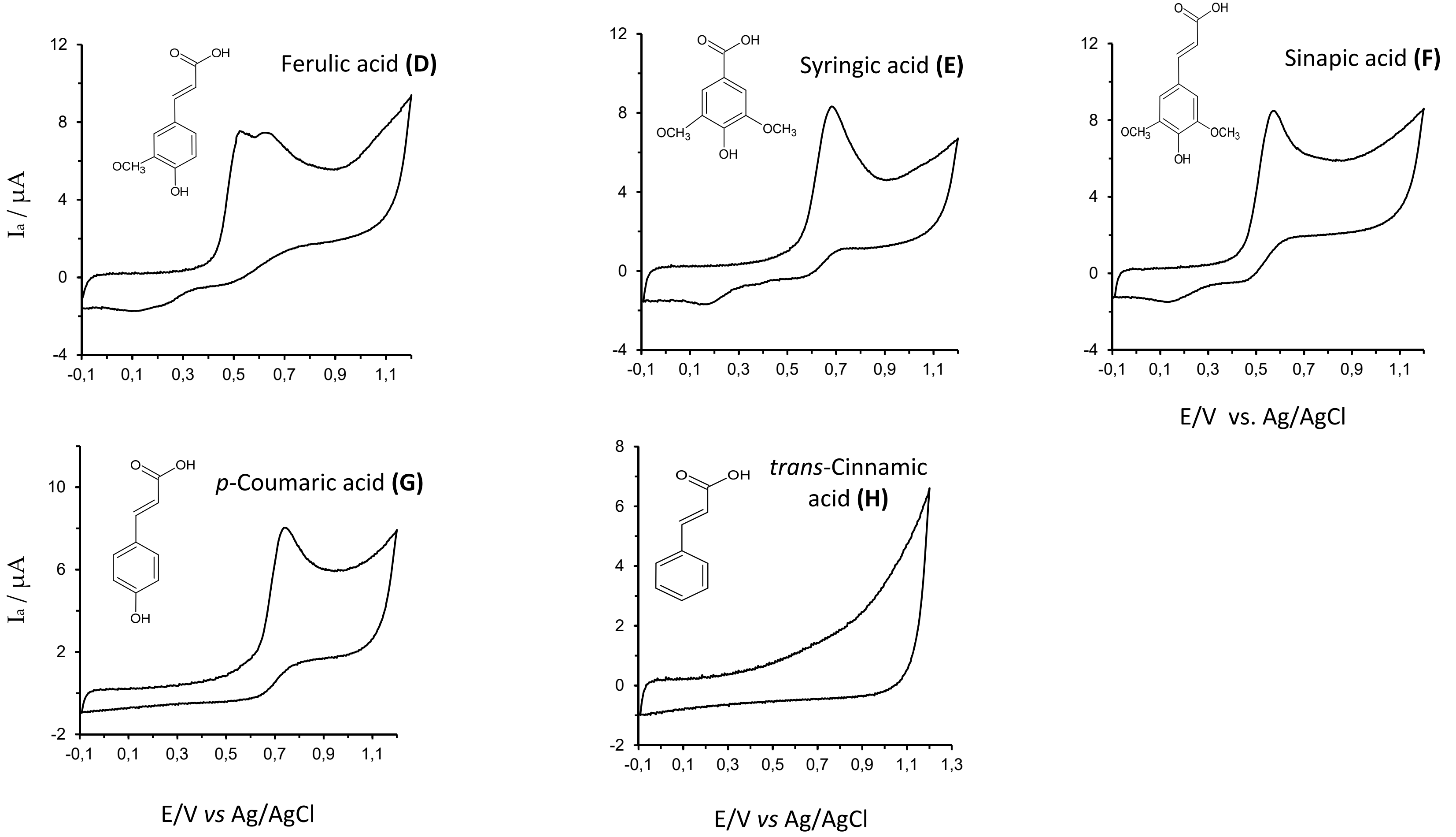

FLAVONOIDS

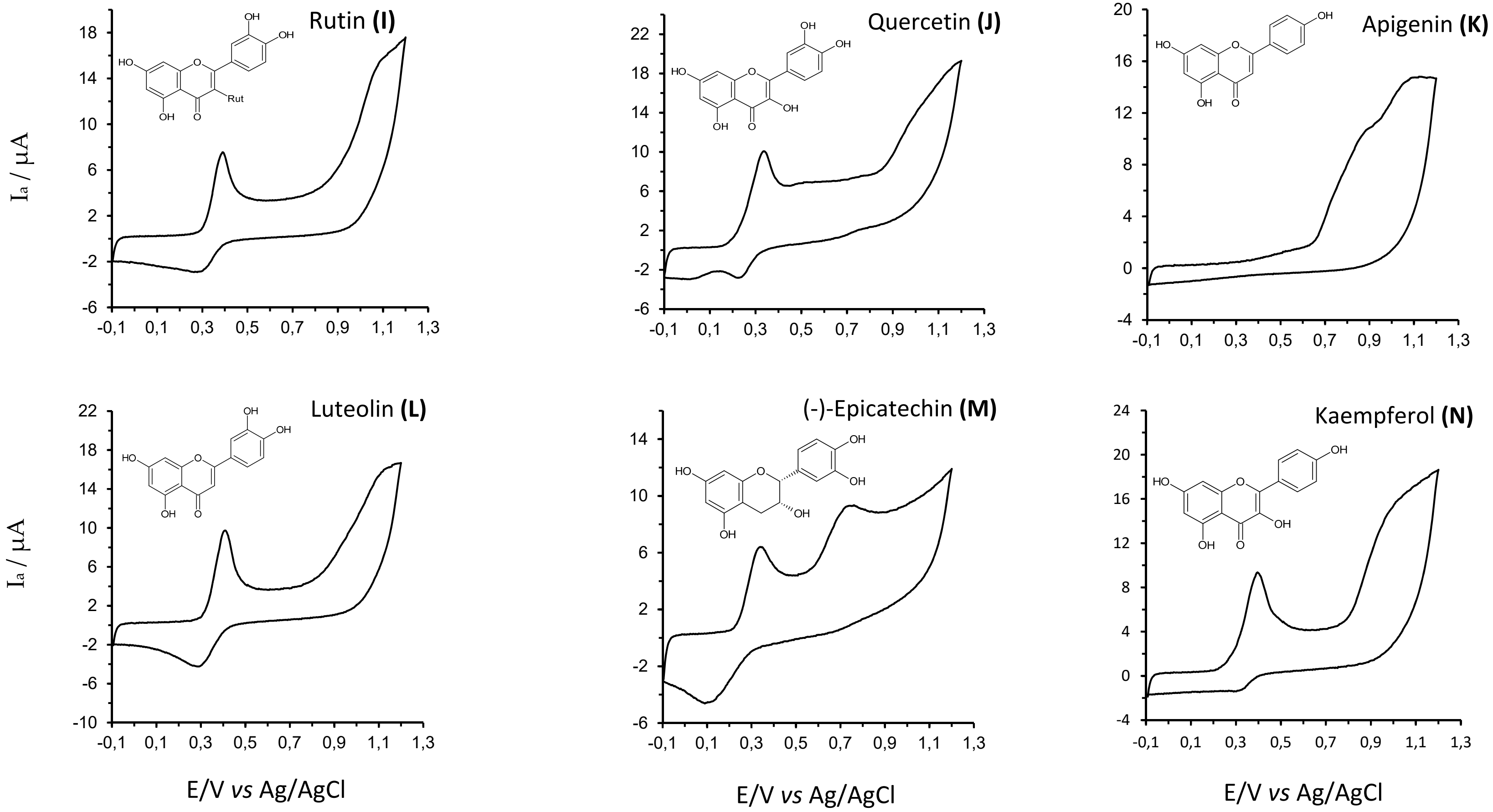

Rutin (I)
Quercetin (J)
Apigenin (K)
Luteolin (L)
(-)-Epicatechin (M)
Kaempferol (N)
Ia / μA
Ia / μA
E/V vs Ag/AgCl
E/V vs Ag/AgCl
E/V vs Ag/AgCl

TROLOX
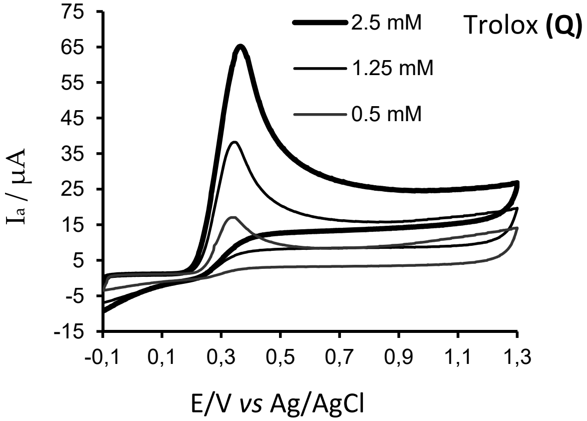75
65
55
45
35
25
15
5
-5
-15
Ia / µA
-0,1
0,1
0,3
0,5
0,7
0,9
1,1
1,3
E/V vs Ag/AgCl
2.5 mM
1.25 mM
0.5 mM
Trolox (Q)

Table S5. Antioxidant, reducing and chelating activities of BW phenolic acids and flavonoids at 1 mM determined by spectrophotometric assays.

Compound/Assay	Antioxidant Activity (mM Trolox) DPPH RSA	Reducing Activity (mM Trolox) FRAP	Chelating Activity (%) FZ
	Phenolic acids		
Vanillic acid	0.82 ± 0.03^{c}	0.05 ± 0.01^{f}	3.55 ± 0.04^{f}
Protocatechuic acid	1.34 ± 0.02^{b}	1.23 ± 0.01^{d}	81.16 ± 0.15^{b}
Caffeic acid	1.31 ± 0.01^{b}	2.24 ± 0.02^{b}	79.08 ± 0.76^{c}
Syringic acid	1.68 ± 0.04^{a}	1.36 ± 0.04^{c}	4.21 ± 0.07^{e}
Sinapic acid	0.84 ± 0.07^{c}	2.40 ± 0.08^{a}	84.12 ± 0.17^{a}
Ferulic acid	0.76 ± 0.01^{d}	0.73 ± 0.02^{e}	0.21 ± 0.04^{g}
p-Coumaric acid	0.72 ± 0.02^{d}	0.04 ± 0.01^{f}	81.12 ± 0.21^{b}
trans-Cinnamic acid	0.14 ± 0.02^{e}	0.02 ± 0.01^{f}	7.11 ± 0.42^{d}
	Flavonoids		
Rutin	1.69 ± 0.02^{c}	1.62 ± 0.03^{d}	72.13 ± 0.16^{c}
Epicatechin	1.37 ± 0.04^{d}	1.60 ± 0.04^{d}	80.26 ± 0.25^{a}
Luteolin	2.07 ± 0.05^{a}	1.40 ± 0.03^{e}	70.94 ± 0.15^{d}
Quercetin	2.09 ± 0.03^{a}	2.58 ± 0.03^{a}	68.96 ± 0.33^{f}
Apigenin	0.11 ± 0.02^{f}	0.02 ± 0.01^{g}	80.29 ± 0.12^{a}
Kaempferol	1.17 ± 0.03^{e}	1.89 ± 0.05^{b}	76.08 ± 0.11^{b}

Results provided by DPPH RSA - DPPH radical scavenging activity assay; FRAP - ferric-reducing/antioxidant power assay; FZ - ferrozine assay. Data are expressed as means ± SD (n = 6). Means in a column related to a respective assay dedicated to phenolic acids or flavonoids, followed by different letters, are significantly different ($p<0.05$) based on the one-way analysis of variance (ANOVA).

References

[1] J. A. Giménez-Bastida, M. Surma, H. Zieliński, *Toxicol In Vitro* **2015**, *29*, 1683–1691.
